# Beyond drive cycles: mapping the intricacies of electric vehicle battery health in diverse environments and driving conditions

**DOI:** 10.1039/d5ra04379d

**Published:** 2025-08-29

**Authors:** Sai Krishna Mulpuri, Bikash Sah, Praveen Kumar

**Affiliations:** a Department of Electronics and Electrical Engineering, Indian Institute of Technology Guwahati Assam 781039 India m.sai@iitg.ac.in; b Chair of Interconnected Automation Systems, University of Siegen Hölderlinstrasse 3, Building A Siegen 57076 Germany Bikash.Sah@uni-siegen.de

## Abstract

As electric two-wheelers become increasingly prevalent in emerging markets, understanding the real-world degradation of their batteries is crucial for ensuring reliability, longevity, and cost-effectiveness. Traditional battery aging studies are heavily based on standard drive cycles that do not capture the variability introduced by diverse user behaviors, regional conditions, and charging habits. This study proposes a comprehensive and adaptable framework for evaluating the end-of-life (EOL) of EV batteries in realistic usage scenarios. The methodology incorporates key metrics of the drive cycle, including acceleration patterns, rest periods, charging frequency, and rates, in multiple daily driving scenarios, three distinct geographic regions, and varying climatic conditions. By linking these operational parameters to electrochemical degradation phenomena, this work reveals the critical influence of user-specific behavior on capacity fade. The insights generated are not only scientifically grounded but also practically relevant to stakeholders across the EV ecosystem. For cell and vehicle OEMs, the findings support region-specific cell design and lifecycle prediction; for EV users, the study offers a clearer understanding of how personal usage affects battery health. Ultimately, this work bridges the gap between lab-scale testing and real-world degradation, paving the way for smarter battery design, personalized usage strategies, and sustainable EV adoption.

## Abstract 2

As electric two-wheelers become increasingly prevalent in emerging markets, understanding the real-world degradation of their batteries is crucial for ensuring reliability, longevity, and cost-effectiveness.

## Graphical abstract

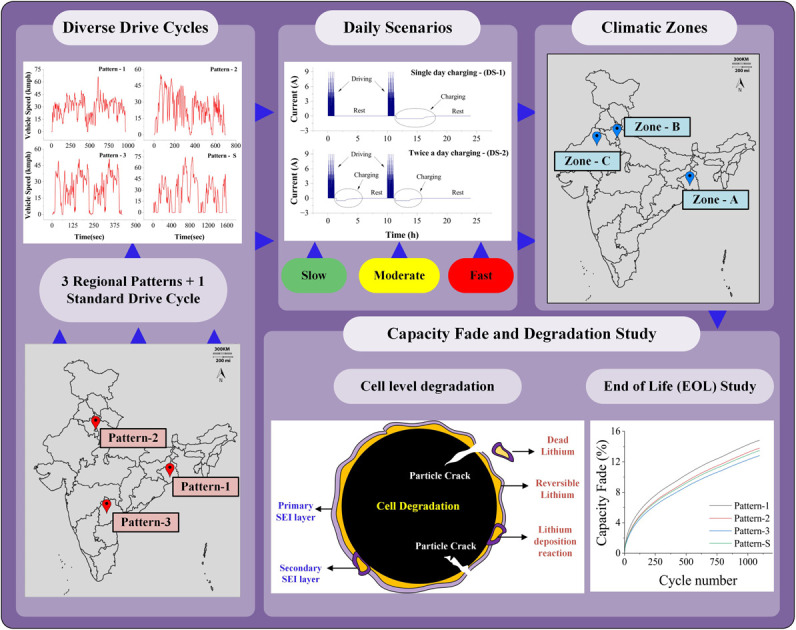

## 1 Introduction

### 1.1 Context and motivation

In recent times, there has been a concerning trend of battery issues in electric vehicles (EVs), with batteries requiring replacement even before reaching their expected end-of-life (EOL) capacity fade of 20%.^1,2^ This poses not only an operational issue, but also a considerable cost-related burden since the battery unit constitutes 35–45% of the overall manufacturing cost of an EV.^3^ Lithium-ion batteries, which dominate the EV market due to their high energy density and cycle life, remain the primary technology for these applications. Researchers continue to face challenges in enhancing battery performance, including improving cycle life, thermal stability, and safety under diverse real-world conditions. Efforts are also directed at developing advanced cathode and anode materials to boost energy density and reduce degradation. Moreover, the limited availability and increasing cost of lithium^4,5^ have driven interest in alternative chemistries such as sodium ion batteries,^6^ solid oxide fuel cells,^7^ and thermal batteries,^8^ which offer diversity in energy storage solutions for similar applications and promise greater sustainability and material diversity for future electric vehicles.

Distinct EVs may exhibit varying EOL periods for the battery packs, even when neighbors use similar models. This discrepancy arises from the influential role of personal usage patterns in the degradation of EV batteries. Another pertinent aspect for vehicle owners is the diversity in charging behavior. Unlike internal combustion engines (ICEs), where the range is independent of fuel refill frequency, EVs face pronounced battery longevity issues. Despite similar driving behaviors and regional and climatic factors for EVs and ICEs, the critical factor of charging a vehicle differs. Consequently, vehicle owners must understand that the EOL of their vehicle's battery pack depends on personal usage patterns, the frequency of recharging, and the rates at which they recharge. Traditionally, original equipment manufacturers (OEMs) primarily assess battery aging based on standard driving profiles provided by manufacturers or recognized drive cycles nationwide (*e.g.*, Worldwide Harmonized Light Vehicle Test Cycle (WLTC),^9^ Urban Dynamometer Driving Schedule (UDDS),^10^ New European Driving Cycle (NEDC),^11^ Indian Driving Cycle (IDC)^12,13^). However, investigating driving style and regional variations is crucial in determining the best- and worst-case scenarios specific to a country or region. Our work emphasizes the need for this generic framework, highlighting the disparity in EOL periods between a standardized drive cycle (WLTC) and regional drive cycles (Pattern-1, Pattern-2, Pattern-3). Building on this analysis, we propose a generic framework for conducting such studies, considering real-time driving behaviours.

The accelerated capacity fade and decline in battery discharge performance in an EV are characterized by two factors: the driving style of the EV user and regional variations in topography and demographics. The driving style of an EV user is reflected in the nature of the drive cycle (*e.g.*, harsh, mild, gentle). It is measured by drive cycle metrics such as kinetic intensity (KI), relative positive acceleration (RPA), and positive kinetic energy (PKE).^14^ It also includes the frequency of vehicle charging and the rates at which the user recharges the vehicle. Secondly, regional variations encompass diverse topographical and demographical conditions due to altitude, latitude, and road profile differences. These variations give rise to distinct driving patterns, impacting acceleration levels, torque requirements, and gradients, particularly in hilly areas. Moreover, the variations in climatic conditions while driving also play a crucial role in impacting battery degradation. This work endeavors to present a meticulous analysis to uncover the underlying reasons behind the accelerated capacity fade and discharge performance deterioration in batteries, thereby highlighting the necessity of incorporating the two factors into aging studies.

### 1.2 Literature review

The studies reported to date remain focused on evaluating battery aging based on standard driving profiles provided by manufacturers or nationwide followed drive cycles.^15–18^ Relying solely on one standard driving pattern to generalize battery fading for an entire country is unreasonable,^19–23^ especially for countries with large land areas and diverse topography and demography. Instead, it is crucial to determine battery fading based on regional drive cycles that consider the specific geographic locations and road profiles.^24^ While some studies do consider different regional drive cycles,^25,26^ they do not fully account for the environmental conditions unique to each region. For instance, tropical and cold countries experience varying climates throughout the year. Temperature plays a pivotal role in battery performance and degradation. At lower temperatures, the electrochemical reaction rates within the battery slow down, resulting in a reduced depth of discharge. Conversely, operating the battery at higher temperatures accelerates the degradation rate as the growth of the solid electrolyte interphase (SEI) layer increases, leading to electrolyte decomposition.^27^ Furthermore, overheating of the battery core causes electrochemical damage,^28,29^ resulting in thermal instability eventually leading to thermal runaway,^30,31^ necessitating efficient thermal management and cooling systems to dissipate heat and mitigate these detrimental effects.^32^ Therefore, it is essential to consider daily temperatures throughout the year or the specific period of interest when analyzing capacity fade. Such considerations are critical to assessing battery performance and degradation under real-world driving conditions accurately.

Drive cycle metrics such as KI, RPA, and PKE and energy exchange at the battery while driving, charging, and resting significantly impact the battery aging and must be explored. While some studies have highlighted that harsher or more aggressive driving behaviors (higher KI, PKE, RPA values) lead to increased battery fading,^16,18,19,25,33^ it is important to differentiate between driving distance and duration. In cases where driving distance remains constant, higher energy exchange (measured in watt-hours) results in greater battery fading. On the other hand, when driving duration is held constant, drive cycles featuring higher KI values are associated with increased battery degradation. However, these statements hold only when we solely consider the driving pattern and overlook the significance of rest periods and charge durations. A daily cycle encompasses more than just driving; it also involves intermediate rest periods^15,33^ when the vehicle is parked and charging periods. Ignoring these essential aspects will lead to an incomplete understanding of battery aging and degradation. It is essential to consider all the operations an EV undergoes in a typical day, including rest^16,18,34^ and charge periods, to gain a deeper insight into the overall battery performance and degradation over time.

The duration of the rest period within a daily cycle is inversely related to the amount of energy discharged. Specifically, higher discharge energies result in longer charging times, reducing the available rest period within a day, and *vice versa* for lower discharge energies. This relationship introduces the concept of calendar aging, wherein the rest period plays a significant role in the overall degradation of the battery over time. Similarly, the impact of charge periods on driving behavior and battery performance is critical. Different charging scenarios with varying charge rates must be studied to understand how charging affects battery performance. Table 1 offers a comprehensive overview of the research conducted by various groups on battery aging studies due to driving behaviour and the associated factors and degradation elements considered in their study. Despite existing studies simulating battery fading behavior based on driving conditions, charge rates, and charging scenarios,^33^ a deeper understanding of cell-level degradation associated with fading is often lacking. Most existing studies lack a comprehensive investigation into SEI layer growth, lithium plating, particle cracking, and the complex interplay between calendar and cycling aging, highlighting the need for deeper exploration of these critical degradation mechanisms. Understanding how degradation occurs during the driving, rest, and charging phases is also essential.

**Table 1:** Overview of existing studies on battery aging due to driving behavior – factors considered/investigated

Standard driving patterns	Regional driving patterns	Different daily scenarios	Different climatic conditions	Impact of charge rates on capacity fade	Entire day analysis	Rest period considered in daily analysis	Reference
Yes	Yes	Yes	Yes	Yes	Yes	Yes	33
Yes	Yes	No	No	No	No	No	25
Yes	No	Yes	Yes	No	Yes	Yes	15
Yes	No	No	No	No	Yes	Yes	16
Yes	No	No	No	No	Yes	No	17
Yes	No	No	Yes	Yes	Yes	Yes	18
Yes	No	No	No	No	No	No	19
Yes	No	No	No	No	No	No	20
Yes	No	No	No	No	No	No	21
Yes	No	No	No	No	No	No	22
Yes	No	No	Yes	No	No	No	23
Yes	Yes	No	No	No	No	No	26

To bridge these gaps and shed light on the complex interplay between driving patterns and battery performance, our study delves into a meticulous examination of regional and standard drive cycles. By meticulously generating diverse regional daily drive cycles from different geographical regions having varied climatic conditions, we aim to unravel the intricate relationship between driving patterns and battery degradation mechanisms. Further, the interplay of rest periods and charge durations on battery degradation is determined by performing the design of experiments involving varying charge rates and frequencies involving rest periods. Through this rigorous and systematic approach, we present a deeper understanding of the degradation mechanisms affecting batteries, thereby providing valuable guidance for developing strategies to enhance battery performance and longevity under real-world driving conditions.

### 1.3 Contributions of this work

This study makes targeted and practical contributions to advancing battery lifespan prediction and customization across the electric mobility ecosystem, impacting cell manufacturers, vehicle OEMs, and end users.

1. *For cell manufacturers*: we introduce a novel framework that links the parameters of real-world usage to the loss of capacity, allowing the precise adjustment of the composition of electrodes and electrolytes according to regional demands. This sets the groundwork for the design of geographically optimized next-generation battery cells.

2. *For vehicle OEMs*: the work offers actionable insights into how diverse driving styles and climatic profiles accelerate or decelerate battery degradation. These insights enable data-driven decisions for warranty planning, battery pack size, and inventory control, while anchoring catalog values in validated, usage-specific degradation trends.

3. *For EV users*: the study explains why two identical EVs driven in different ways, or even similarly but charged differently, can reach end of life (EOL) at dramatically different times. By revealing how charging frequency and rates, daily drive routines, and environmental conditions interplay in shaping battery aging, this work equips users with the knowledge to extend battery life through informed behavioral adjustments.

Technically, this work is among the first to simulate battery degradation by combining three regional contexts, multiple climatic conditions, weekday behavioral variations, and distinct driving behaviors, providing a scalable model for EOL prediction. The modularity of the framework allows easy extension to other geographies or usage patterns, making it adaptable for global applications. By integrating diverse driving behaviors, climatic conditions, and electrochemical analysis, this research bridges a critical gap between laboratory aging tests and real-world battery degradation, setting a new benchmark for how EV battery aging should be studied, understood, and mitigated.

## 2 Methodology

To estimate the impact of driving behaviors, regional variations, daily scenarios, and climatic conditions on battery capacity fade, this paper assumes different driving behaviors, three regional variations, two man-made driving scenarios on weekdays, and three temperature zones. It should be noted that additional behaviors, regions, scenarios, and climatic zones could be developed to represent the operating conditions of EVs in specific regions, and similar analyzes can be performed. A thorough understanding of the underlying phenomena and electrochemical processes is essential to accurately relate the impact of different parameters on cell capacity fade.

### 2.1 Need for real-world driving cycles

Real-world driving cycles play a pivotal role in accurately assessing battery performance for driving behaviors. Unlike standardized drive cycles, which offer a limited representation of regional driving behaviors, real-world conditions exhibit significant variations based on traffic patterns, road conditions, charging patterns, and driving habits. These dynamic factors, such as sudden start-stops, frequent accelerations, and decelerations in response to traffic conditions, demand diverse power outputs from the battery, rendering the current variations far from constant. Standard driving cycles fail to capture these intricate dynamics, leading to an underestimation of capacity fade compared to real-world scenarios. The absence of accurate representation in standard cycles overlooks the true degradation process.

#### 2.1.1 Drive cycle metrics and their significance

To quantify the characteristics of the dynamics in a driving cycle, drive cycle metrics serve as essential tools. These metrics comprehensively assess driving patterns by capturing information on velocity, distance covered, acceleration, and more. Key parameters among these standard drive cycle metrics are KI, RPA, and PKE. These metrics play a crucial role in understanding the harshness or gentleness of a driving cycle, shedding light on how vehicle speed fluctuates and the energy involved throughout the driving process. Kinetic intensity (KI) is a crucial drive cycle metric that sheds light on the dynamic behavior of vehicles during their operational cycles. It quantifies the overall changes in kinetic energy and serves as a time-averaged measure of the intensity of vehicle dynamics. Comprising two essential metrics, KI is the ratio of characteristic acceleration (*a*_C_) to the square of the aerodynamic speed (*v*_aerodynamic_^2^), as shown in the eqn (1).^14^ The first metric, characteristic acceleration, measures the inertial work required to accelerate or lift the vehicle per unit mass and distance over the cycle (eqn (2)). It captures the positive part of specific kinetic and potential energy per distance associated with the vehicle's movement. The second metric, the square of the aerodynamic speed, gauges the ratio of the overall average cubic speed to the average speed (eqn (3)). This element directly accounts for the influence of aerodynamics on vehicle performance.1
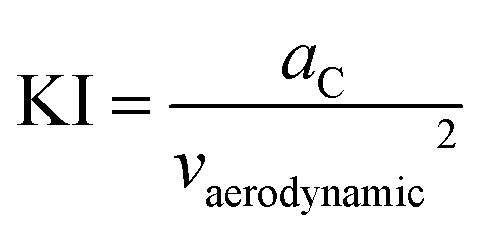
2
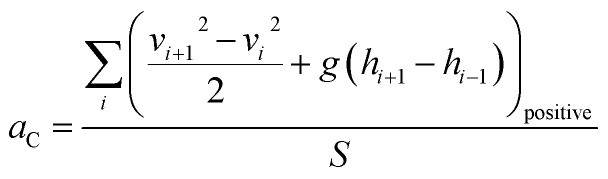
3
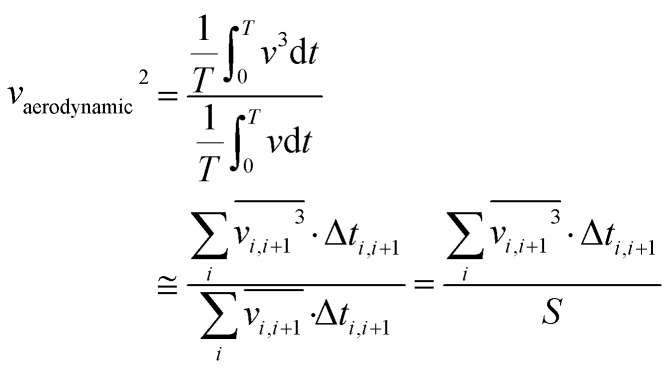
where ‘*v*_*i*_’ is the vehicle speed at *i*th instant, ‘*g*’ is the acceleration due to gravity, ‘*h*’ is the elevation, ‘Δ*t*’ is the successive time interval between two points, ‘*T*’ is the total time spent for driving, and ‘*S*’ is the total distance travelled. KI is highly valuable for assessing the intensity of driving conditions and evaluating the energy demands on vehicles. In particular, higher KI values indicate aggressive driving behavior, characterized by rapid and frequent speed changes. Conversely, lower KI values suggest a smoother and more consistent driving behavior, involving fewer variations in kinetic energy. Moreover, KI enables a quantitative characterization and comparison of different drive cycles, such as urban, highway, or specific test cycles, allowing for a comprehensive understanding of their varying levels of intensity and aggressiveness. By evaluating the energy basis using the characteristic acceleration and aerodynamic speed metrics, KI provides a holistic representation of the duty cycle's impact on road loads.

Relative positive acceleration (RPA) measures the positive acceleration experienced by the vehicle during the drive cycle, normalized by the average speed during the travel. It is calculated by taking the speed differences between consecutive instants at regular intervals, typically every second, and filtering out vehicle speed changes on time scales less than two seconds. The result measures specific power averaged by time, considering only positive acceleration instances (eqn (4)).^14^ Here, ‘*v*_*i*_’ is the vehicle speed corresponding to *i*th data point, ‘Δ*t*’ is the successive time interval between two data points, and the numerator contains the summation of only the positive acceleration terms. High RPA values indicate frequent and significant positive accelerations during the drive cycle, which implies a more aggressive driving style with frequent speed changes, indicating more stop-and-go traffic conditions. Lower RPA values indicate a smoother, more consistent driving style with less frequent positive accelerations. RPA is particularly useful in assessing the dynamics of urban driving, where traffic congestion and frequent stops can be observed.4
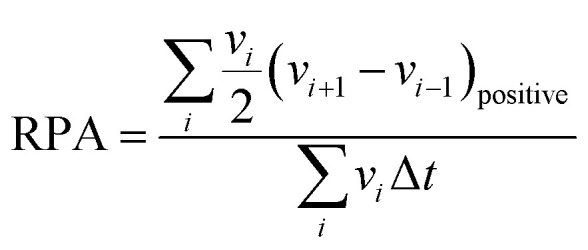


Positive kinetic energy (PKE) is a metric that quantifies the positive kinetic energy added to the vehicle during the drive cycle, normalized by the total distance traveled. The calculation of this metric involves computing the ratio of the sum of positive differences in vehicle speeds between consecutive data points, which signifies instances of individual positive acceleration, typically measured at regular time intervals, to the total distance covered during the given period (eqn (5)).^14^ Here ‘*v*_initial_’ and ‘*v*_final_’ are the initial and final speeds, respectively, during the individual positive acceleration instances, and this summation is divided by the total distance travelled, ‘*S*’. High PKE values indicate that the vehicle undergoes significant kinetic energy changes, experiencing frequent decelerations. A drive cycle with high PKE values often reflects a driving pattern with many speed changes, such as city driving or hilly terrain. Lower PKE values suggest a steadier, more consistent driving style with fewer speed changes.5
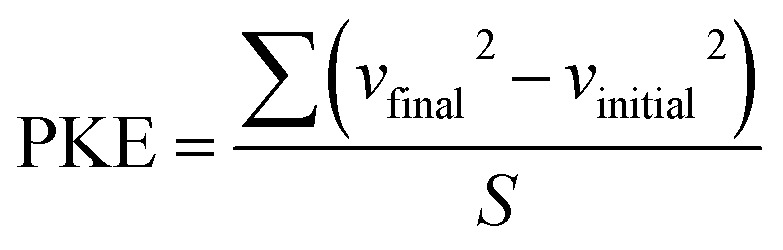


#### 2.1.2 Choice of regions, driving behaviours and their significance

Having highlighted the need for real-world driving cycles and their associated metrics, the subsequent subsection discusses the process of choosing diverse drive cycle regions, characterized by different metrics indicating distinct driving patterns and behaviors. This can effectively capture critical factors such as sudden start stops, frequent accelerations and decelerations, and varying power demands from the battery during driving.

**Fig. 1:**
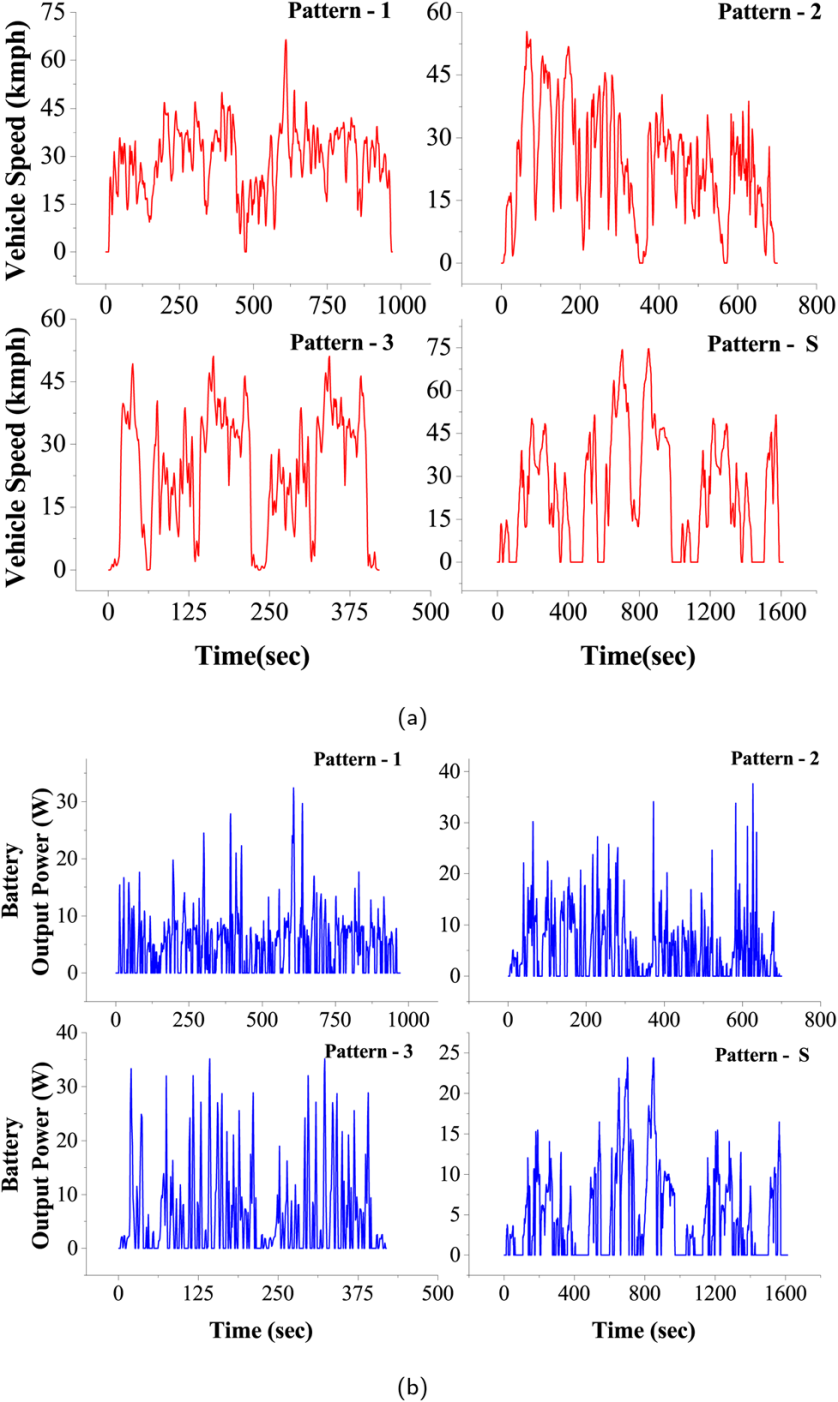
(a) Vehicle speed (in kmph) *vs.* time (in seconds) drive profiles for four driving patterns, including three regional patterns and one standard pattern. (b) Battery output power (in W) *vs.* time (s) profiles corresponding to the drive profiles.

To comprehensively assess battery capacity fade behavior, a case study in three distinct regions in India is performed: Eastern India – Panskura, West Bengal, Northern India – Kapashera, New Delhi, and Southern India – Hyderabad, Telangana. Our team at E-Mobility Lab, IIT Guwahati, gathered extensive data, covering over 150 km of driving in each region over two weeks, capturing diverse traffic and geographical conditions. The collected data was meticulously analyzed to generate corresponding real-world driving cycles, following a detailed process outlined in ref. 35. A standard drive cycle, WLTC-class2, was also included for comparative analysis with the diverse driving profiles.^9^ The WLTC is a global standard test cycle adopted by many countries, including China, Japan, the United States, India, and the European Union, among others.^36^ In this study, the four driving patterns observed in Panskura, New Delhi, Hyderabad, and WLTC, as shown in Fig. 1a, are referred to as Pattern-1, Pattern-2, Pattern-3, and Pattern-S, respectively, in further discussions.

The key parameters, such as average speed and maximum speed for all considered driving patterns, were derived from the vehicle speed *vs.* time data (Fig. 1a). The critical drive cycle metrics were calculated using empirical relations from eqn (1), (4), and (5). These statistical outcomes are described in the Table 2 demonstrates that all three real-world driving profiles exhibit higher KI, PKE, and RPA values compared to the standard driving profile, WLTC-class2. These findings highlight the significant discrepancies between standard driving profiles and actual driving conditions, emphasizing the need for real-world driving cycles to accurately estimate battery capacity. The power demanded from the battery output was determined based on the distinct driving patterns. For this study, the LGM50 21 700 cylindrical cell with a nominal voltage of 3.6 V and a nominal capacity of 5 Ah was chosen. The detailed process of extracting battery power from vehicle speed and time profiles is outlined in ref. 35. The power demands for the four driving profiles are presented in Fig. 1b.

**Table 2:** Inclusive critical drive cycle metrics, average speed, and maximum speed information for all considered driving patterns in the study

Pattern	KI (1/m)	PKE (m/s^2^)	RPA (m/s^2^)	Maximum speed (kmph)	Average speed (kmph)
Pattern-1	0.0023	0.41	0.018	66.47	28.70
Pattern-2	0.0039	0.67	0.057	55.42	23.56
Pattern-3	0.0046	0.81	0.083	51.13	23.66
Pattern-S	0.0010	0.29	0.010	74.70	24.51

**Fig. 2:**
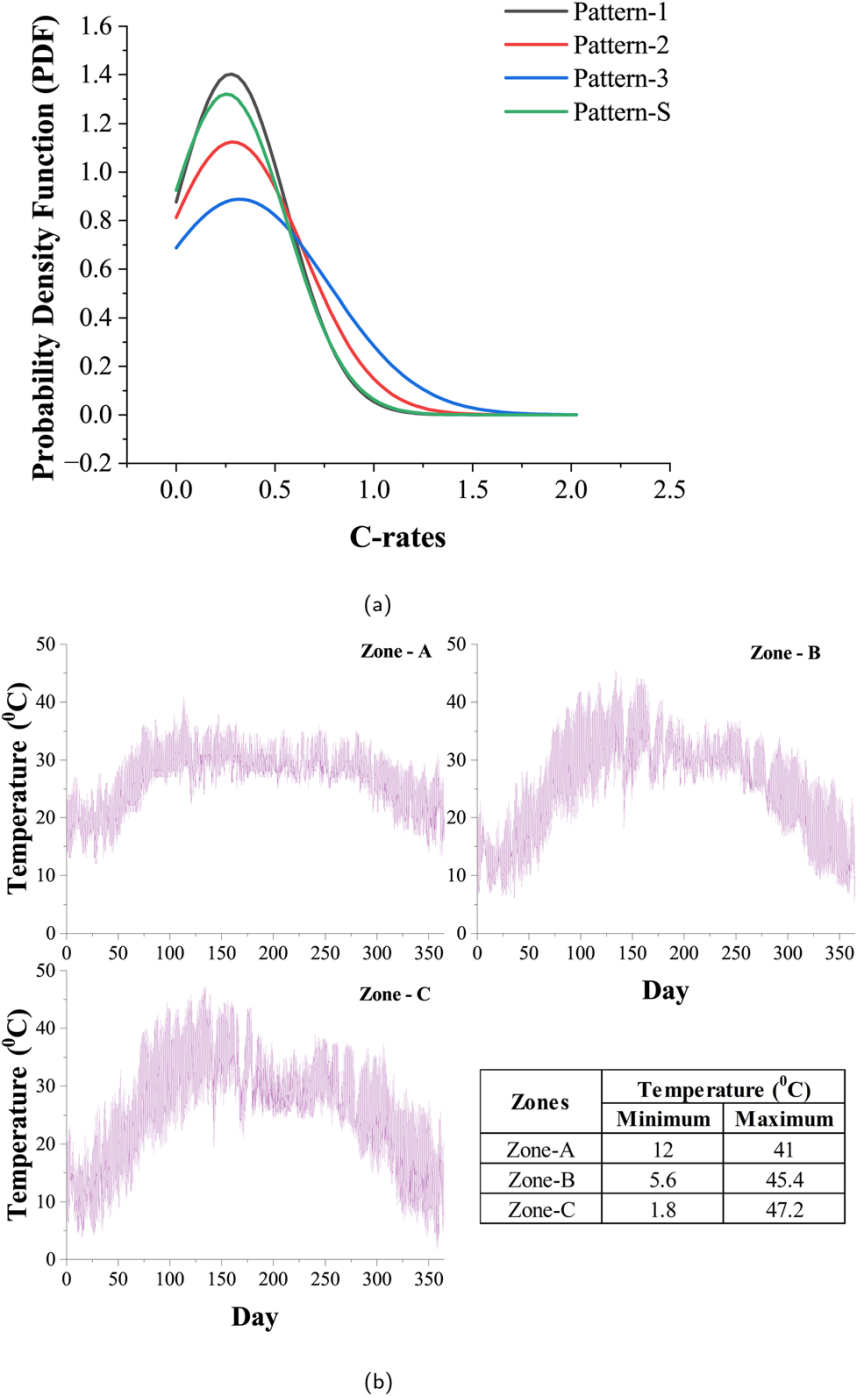
(a) Normal probability density function (pdf) distribution of different C-rates across four driving patterns. (b) Temperature profiles of three climatic zones across India for the year 2022 alongside their respective extreme temperature limits.

Furthermore, Fig. 2a illustrates the probability density functions (PDFs) for C-rates associated with four distinct driving patterns for a single trip, offering a clear visual representation of the variations in driving styles and the corresponding demands they place on a vehicle's battery system. Here, the C-rate is the measure of the rate at which a battery is charged/discharged with respect to its capacity. For instance, a 1C rate corresponds to a full charge or discharge in 1 hour. Each pattern presents a unique distribution that characterizes the frequency and intensity of the power requirements during a single driving trip.

Pattern-1, denoted by the black curve, has a PDF that peaks at lower C-rates, along with a kinetic intensity value of 0.0023 m^−1^, indicating a driving style with moderate energy demands, reflective of a cautious and steady driving style maintaining consistent speeds and avoiding sudden accelerations. Similarly, Pattern-S, depicted by the green curve, also has the lowest kinetic intensity value of 0.0010 m^−1^, with its peak sharply centered at the lowest range of C-rates. This distribution represents a conservative driving pattern, resembling a controlled testing scenario like a standard drive cycle in a laboratory setting, closer to an eco-driving style. The driving style associated with Pattern-S is gentle on the battery, potentially resulting in minimal wear and the longest possible battery life.

On the contrary, Pattern-2, illustrated by the red curve, shows a PDF with a higher spread of C-rates and further has the kinetic intensity of 0.0039 m^−1^. The broader distribution and shift towards higher C-rates imply a more dynamic and varied driving style. This pattern is indicative of a driving style experiencing more frequent stop-and-go conditions or having a tendency to accelerate more aggressively. Such a driving pattern could result in increased power demands. Likewise, Pattern-3, represented by the blue curve, also has the highest kinetic intensity value of 0.0046 m^−1^, with its PDF extending further into the higher C-rate region. This suggests an even more aggressive driving style than Pattern-2, characterized by frequent hard accelerations and high-power demands. This driving behavior may lead to faster battery consumption and could necessitate more frequent charging intervals.

These findings highlight the substantial variation in power demands across different driving cycles, emphasizing the significance of capturing such real-world dynamics in battery performance assessments. The diversity in driving behaviors influences how the battery discharges, which is crucial for designing batteries and battery management systems that cater to various use cases.^37^

### 2.2 Different climatic conditions

This section discusses the methodology employed to investigate the effect of temperature on battery capacity fade in everyday driving scenarios for EVs, considering driving conditions, battery technology, and temperature as critical factors.^26,38,39^ Temperature significantly influences battery life, with high and low temperatures adversely affecting performance.^40^ To comprehensively understand temperature effects, subjecting the battery to a constant temperature would provide limited insights, as real-world ambient temperatures are dynamic. Thus, this study adopts an approach involving hourly temperature variations to capture the impact realistically. Three distinct temperature zones, Eastern India – Kolkata (Zone-A), Northern India – Delhi (Zone-B), and Western India – Rajasthan (Zone-C), were selected based on their diverse temperature variations throughout the year, aiming to assess how different temperature profiles impact battery performance.

Hourly temperature data for these regions for the entire year 2022 were collected.^41^ These regions were chosen for their significant temperature spreads and extreme conditions experienced during the year (Fig. 2b). Churu experiences extreme temperatures ranging from 1.8 °C (extreme low) to 47.2 °C (extreme high), followed by Delhi with extreme temperatures of 5.6 °C (extreme low) and 45.4 °C (extreme high), both posing considerable challenges for battery performance. Kolkata, on the other hand, depicts a more moderate temperature profile with extreme temperatures ranging from 12 °C to 41 °C. The impact of these diverse temperature profiles on battery performance is further discussed and analyzed in subsequent sections, aiming to provide valuable insights into battery behavior under varying temperature conditions.

### 2.3 Daily scenarios

In this section, we address the significance of considering a typical daily cycle to accurately estimate an EV battery's performance. A comprehensive analysis requires accounting for an EV's various stages during the day, encompassing driving, resting, and charging periods. Accordingly, two distinct scenarios are considered: Daily Scenario-1 (DS-1), representing a typical work cycle, where the EV experiences morning driving, rests during the day while parked, evening driving returning from work, and charges at home till reaching full capacity; and Daily Scenario-2 (DS-2), simulating driving to work, charging at work until full capacity, resting until evening, driving back from work to home, and then charging at home until reaching full capacity. The charging method employed in this study follows the standard Constant Current Constant Voltage (CCCV) protocol, renowned for its energy-efficient approach to charging.^42,43^

**Fig. 3:**
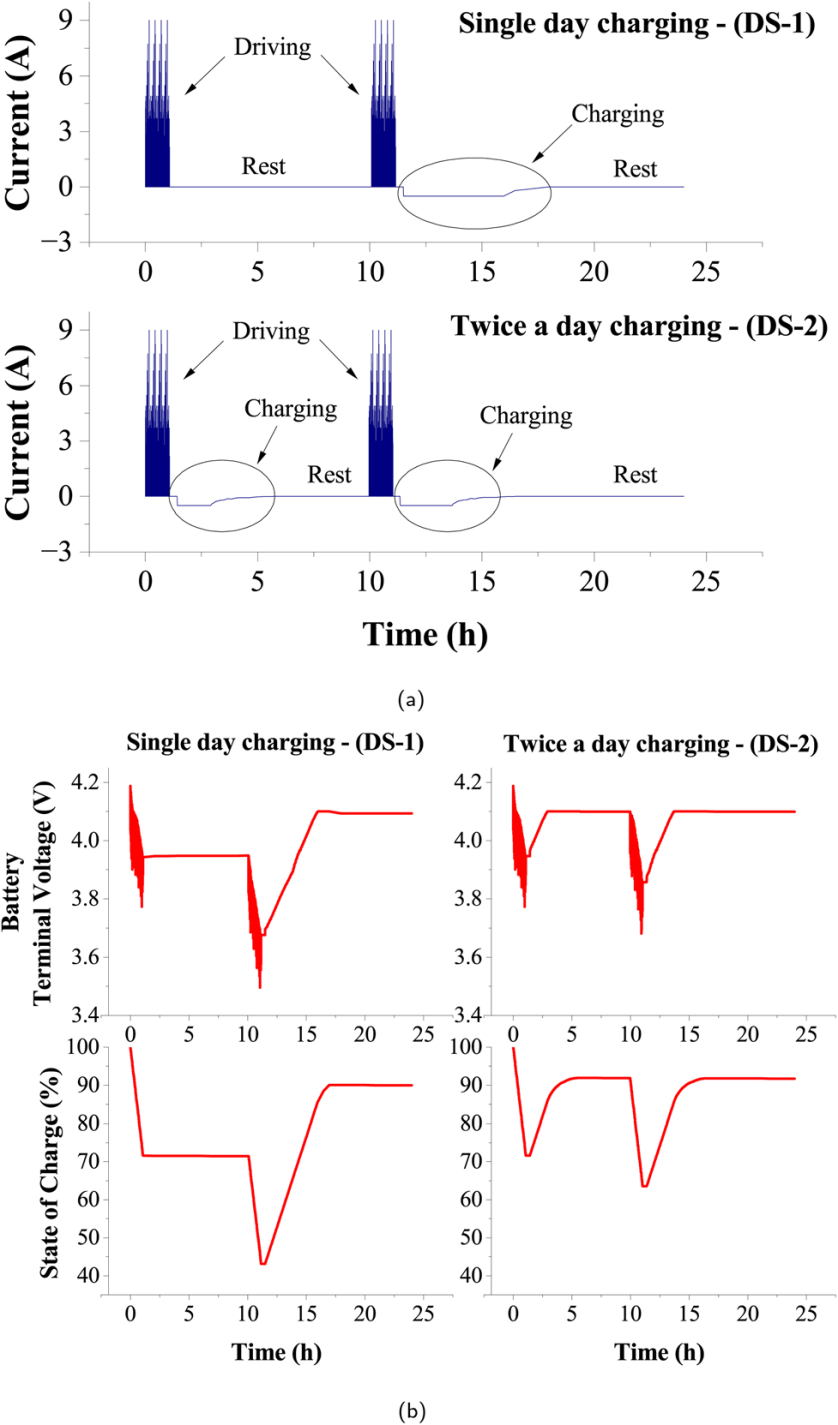
(a) Two daily scenarios illustrate all the possible phases in an EV's daily cycle. (b) State of charge (SOC) and terminal voltage profiles vary daily for the two daily scenarios.

These scenarios aim to capture the dynamics of single-day charging and twice-a-day charging conditions as shown in Fig. 3a. For a chosen Pattern-1, corresponding State of Charge (SOC) and terminal voltage profile variations in a day for a fully charged battery subjected to the two scenarios DS-1 and DS-2 are illustrated in Fig. 3b. Furthermore, within these scenarios, we explore the impact of multiple charge rates during the charging stages to assess their influence on battery performance. By varying charge rates, we gain insights into how different charging speeds affect battery behavior under different scenarios.

### 2.4 Design of experiments

The investigation into the intricate relationship between charge rates and periods, driving patterns, and resting periods is of utmost importance to accurately assess battery capacity fade. This section is dedicated to presenting our meticulously designed experiments in this paper, aimed at unraveling the complexities associated with EV driving behaviors and their impact on battery performance.

**Fig. 4:**
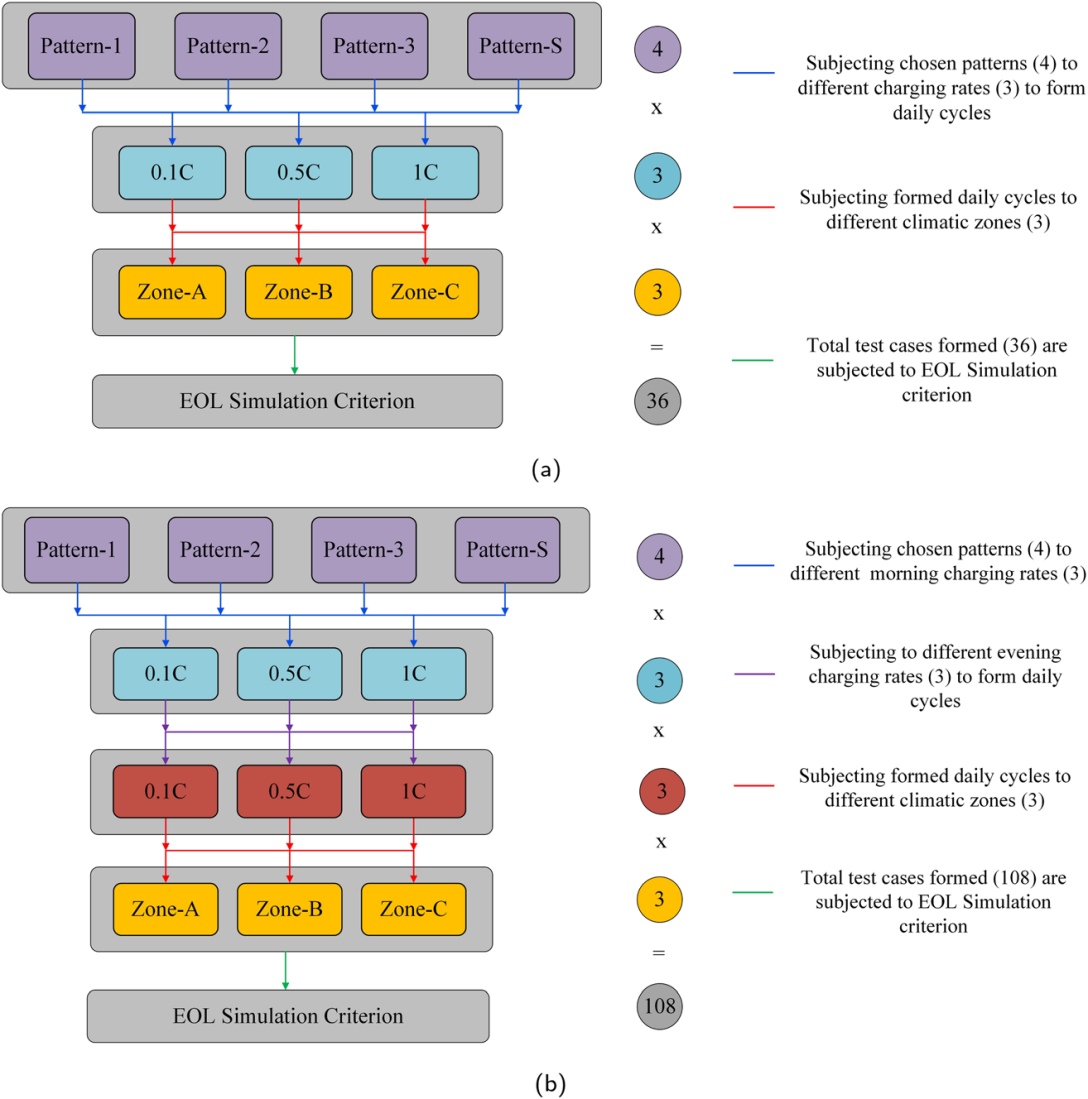
Comprehensive simulation cases were designed, exploring the influence of driving patterns, charge rates, and climatic conditions in (a) daily scenario-1 and (b) daily scenario-2.

Typically, the life cycle of an EV battery pack is measured in terms of years or kilometers driven. In this work, we consider a daily driving distance of 60 km, along with charging and rest periods within a day, subjecting the battery to an end-of-life (EOL) simulation. For two-wheelers, manufacturers typically provide a 3 year EOL period.^44–46^ Battery EOL is defined when the capacity reaches 80% of its nominal capacity.^1,2^ Therefore, in this study, we adopt the EOL criteria as the earlier occurrence of a 3 year daily cycle simulation or the capacity fade to 20% of its original capacity. Consequently, an EV would travel approximately 65 700 km in 3 years if the 20% fade has not yet occurred. However, real-world driving cycles may yield different life expectancies compared to manufacturer-specified values since they often test battery packs using standard driving profiles, less kinetic-intensive than real-world profiles. Additionally, they do not consider the entire daily cycle with varying charge rates and temperature profiles. As discussed, the two designed scenarios encompass driving cycles with accompanying rest and charging stages. In this study, the four selected driving patterns (Pattern-1, Pattern-2, Pattern-3, Pattern-S) are simulated with charge rates of 0.1C (slow charging), 0.5C (moderate charging), and 1C (fast charging). Each daily cycle generated is subjected to the EOL simulation criteria. Furthermore, the designed set of test cases, obtained through the combination of driving patterns and charge rates for both scenarios, is subjected to three chosen temperature zones (Zone-A, Zone-B, Zone-C) until the EOL simulation. In Scenario 2, the two charging stages in a day undergo different combinations of the chosen charge rates (0.1C, 0.5C, and 1C). The total simulation cases designed for DS-1 and DS-2 are illustrated in Fig. 4a and b, respectively. The simulation-based experiments performed in this work are carried out on open-source battery modelling software, Python Battery Mathematical Modelling (PyBaMM).^47^ PyBaMM facilitates continuum model simulation with advanced electrochemical models like DFN, SPM, and SPMe. It also integrates submodels for degradation mechanisms like SEI growth, lithium plating, and particle cracking. PyBaMM's capability to couple these mechanisms enables effective observation of various degradation patterns and electrochemical behaviors.^48–50^

## 3 Background synthesis and methodology formulation

### 3.1 Mathematical modelling of electrochemical model of cell

To capture various electrochemical phenomena within a cell, mathematical models are utilized to replace time-consuming real-time experiments. Moreover, for designing efficient battery management systems, mathematical models are vital. The electrochemical models (EM) and equivalent circuit models (ECM) are the most commonly used models for developing the mathematical model of the cell. ECMs are developed using resistors, capacitors, and voltage sources to form a circuit network, providing a simplistic framework for modeling battery behavior.^51–53^ However, they come with inherent limitations. ECMs often fail to capture the nuanced complexities of battery performance due to their oversimplified nature, rendering them unable to precisely replicate the intricate electrochemical processes occurring within the cell.

In contrast, electrochemical models offer a more comprehensive approach. Models such as Doyle-Fuller Newman (DFN)^54^ and Single Particle Model with Electrolyte (SPMe)^55^ utilize a sophisticated set of coupled nonlinear differential equations to describe the intricate phenomena such as diffusion of solutes in electrode material, chemical reaction rates, electrode transport properties, capacity fade and cell degradation within the battery cell. By incorporating detailed insights into electrode and interfacial microstructures, along with fundamental electrochemical principles, these models can accurately depict the behavior of the battery under various operating conditions. Despite their computational complexity and memory requirements, electrochemical models stand out for their ability to provide a precise representation of battery behavior, making them indispensable tools for understanding and optimizing battery performance in practical applications. The Doyle-Fuller Newman (DFN) model, a standard continuum mathematical model for lithium-ion batteries, is renowned but computationally intensive due to its complex nonlinear partial differential equations. To address this challenge, simpler models like the Single Particle Model (SPM) are employed, yet they often lack accuracy in voltage prediction without correction terms. Consequently, this study opted for the Single Particle Model with electrolyte (SPMe) due to its enhanced accuracy compared to the basic SPM. This section outlines the fundamental modeling and governing equations of the SPMe model. For in-depth derivations and equations, readers are directed to ref. 55.

The concentration of lithium in the positive and negative particles, as well as the lithium ion concentration in the three regions of the electrolyte, are governed by three linearly independent partial differential equations (PDEs) (eqn (6a)–(6c)).^55^ The boundary conditions and the initial conditions for these three governing equations are defined from eqn (6d) to (6j). The subscripts “n”, “s”, and “p” represent variables associated with the negative electrode, separator, and positive electrode, respectively. In addition, the subscripts “e” and “s” are appended to indicate electrolyte variables and solid-phase variables, respectively. The terminal voltage (eqn (6k)–(6r)) is determined by solving these equations and can be calculated using a simple and easily understandable algebraic expression.^55^ These equations form the basis of the Single Particle Model with electrolyte (SPMe) utilized in this study (Table 3).

**Table 3:** Symbols used in the modelling of the SPMe model

Symbol	Parameter
*L*_n_	Negative electrode thickness
*L*_s_	Separator thickness
*L*_p_	Positive electrode thickness
*R*_n_	Radius of −ve active material particles
*R*_p_	Radius of +ve active material particles
*ϕ*	Electric potentials
*j*	Current densities
*c*	Lithium concentrations
*N*	Molar fluxes
*x*	Microscopic spatial variable
*r*	Macroscopic spatial variable

Governing equations6a
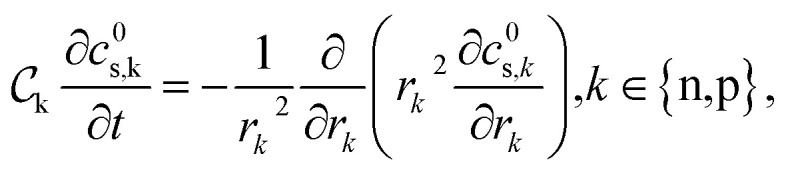
6b
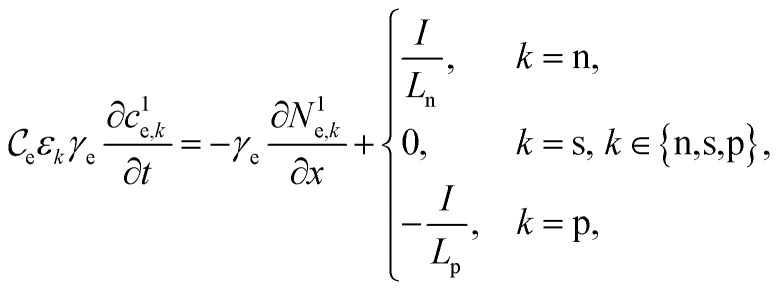
6c
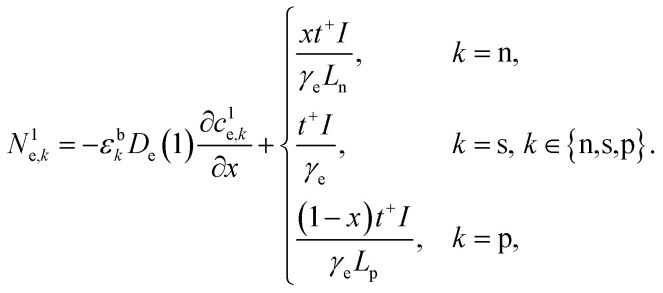


Boundary conditions6d
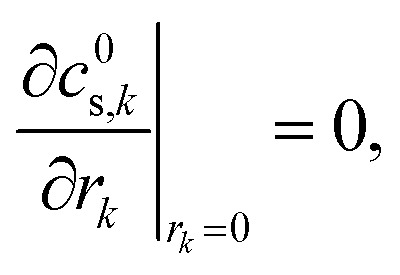
6e
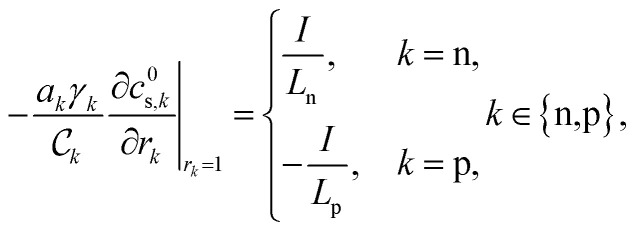

6f *N*^1^_e,n_|_*x*=0_ = 0, *N*^1^_e,p_|_*x*=1_ = 0,

6g *c*^1^_e,n_|_*x*=*L*_n__ = *c*^1^_e,s_|_*x*=*L*_n__, *N*^1^_e,n_|_*x*=*L*_n__ = *N*^1^_e,s_|_*x*=*L*_n__,

6h *c*^1^_e,s_|_*x*=1−*L*_p__ = *c*^1^_e,p_|_*x*=1−*L*_p__, *N*^1^_e,s_|_*x*=1−*L*_p__ = *N*^1^_e,p_|_*x*=1−*L*_p__.

Initial conditions

6i *c*^0^_s,*k*_(*r*_*k*_,0) = *c*_*k*,0_, *k* ∈ {*n*,*p*},

6j *c*^1^_e,*k*_(*x*,0) = 0, *k* ∈ {*n*,*s*,*p*}.

Terminal voltage6k
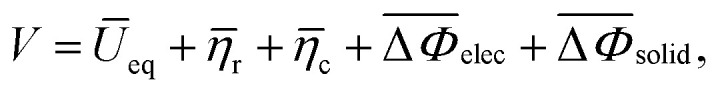
where6l
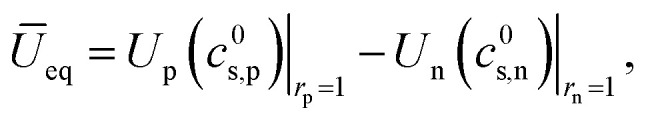
6m
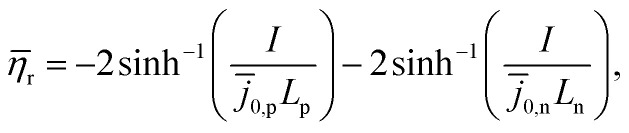
6n
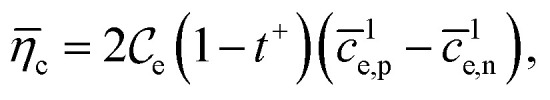
6o
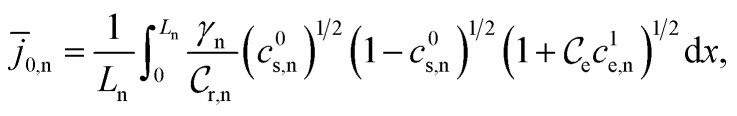
6p
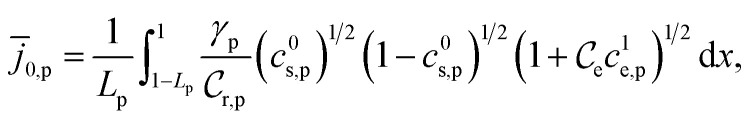
6q
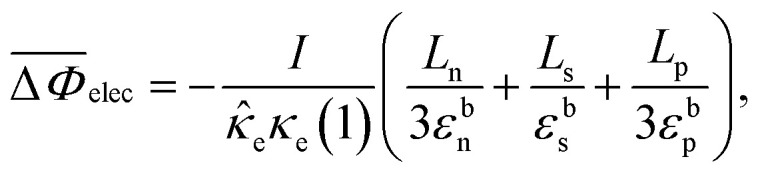
6r
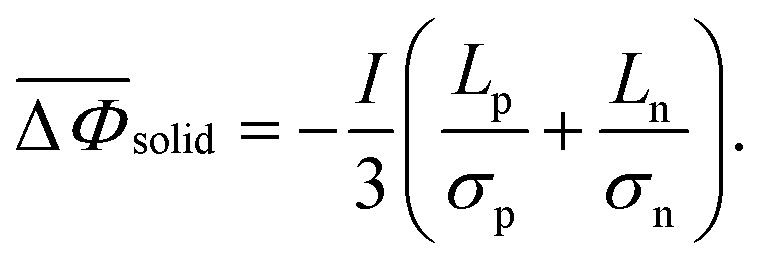


#### 3.1.1 Electrode parameters

The modeling equations for the electrode properties (eqn (7) and (8)) utilized in this work are presented in this subsection.^49^ The open-circuit potential curves 
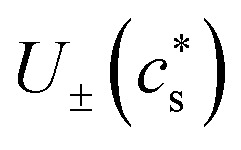
 were initially measured by Chen *et al.*^56^ at 25 °C. The measurements were conducted using a three-electrode cell setup for both electrodes. Chen *et al.* observed considerable hysteresis in the graphite + SiO_*x*_ negative electrode. The other electrode parameters are taken from Chen *et al.* and they are presented in Table 4.

**Table 4:** Electrode parameters used in this work^49^

Symbol	Definition	− Electrode	+ Electrode
*A*	Total planar electrode area, m^2^	0.1027	0.1027
*a*_±_	Surface area to volume ratio, m^−1^	3.84 × 10^5^	3.82 × 10^5^
*c*_m±_	Maximum Li^+^ concentration, mol m^−3^	33 133	63 104
*c*_0±_	Initial Li^+^ concentration, mol m^−3^	29 866	17 038
*D*_±_	Li^+^ diffusion coefficient at 25 °C, m^2^ s^−1^	3.3 × 10^−14^	4 × 10^−15^
*E*_*D*±_	Activation energy for Li^+^ diffusion, J mol^−1^	30 300 (ref. 57)	25 000 (ref. 58)
*E*_*k*±_	Activation energy for rate constant, J mol^−1^	35 000	17 800
*k*_±_	(De)intercalation rate constant at 25 °C, m s^−1^	2.12 × 10^−10^	1.12 × 10^−9^
*r*_±_	Electrode particle radius, m	5.86 × 10^−6^	5.22 × 10^−6^
*δ*_±_	Electrode thickness, m	8.52 × 10^−5^	7.56 × 10^−5^
*ε*_e_	Electrolyte volume fraction	0.25	0.335
*ε*_a_	Active material volume fraction	0.75	0.665
*σ*_±_	Electrode conductivity, S m^−1^	215	0.18

The temperature-dependent parameters solid-state diffusion coefficients 
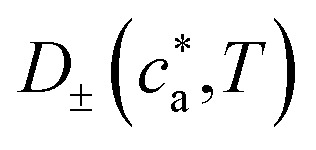
 and effective conductivity *k*_±_(*T*) are assumed to have Arrhenius temperature dependence:7
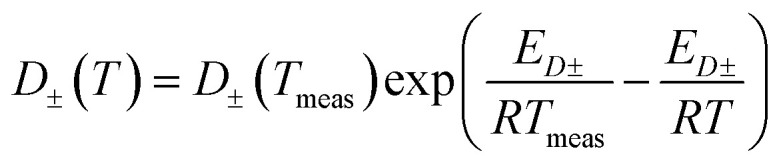
8
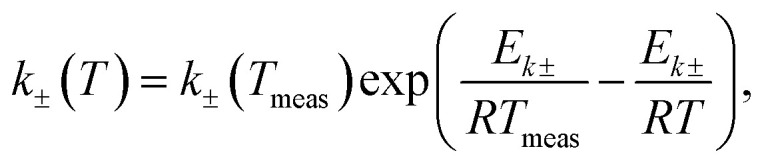
where *E*_*D*±_ and *E*_*k*±_ are activation energies and *T*_meas_ is 298.15 K (25 °C).

#### 3.1.2 Electrolyte parameters

This subsection presents the modeling equations utilized in this work for the electrolyte properties (eqn (9)–(13)).^49^ The effective conductivity *κ*_eff_(*c*_e_,*T*) and diffusion coefficient *D*_eff_(*c*_e_,*T*) of electrolyte occupying volume fraction *ε* are related to the corresponding values *κ*(*c*_e_,*T*) and *D*_e_(*c*_e_,*T*) in pure electrolyte by

9 *κ*_eff_(*c*_e_,*T*) = *ε*_*e*_^1.5^*κ*(*c*_e_,*T*) and *D*_eff_(*c*_e_,*T*) = *ε*_e_^1.5^*D*_e_(*c*_e_,*T*).

Both *κ*(*c*_e_,*T*) and *D*_e_(*c*_e_,*T*) have an Arrhenius temperature dependence:10
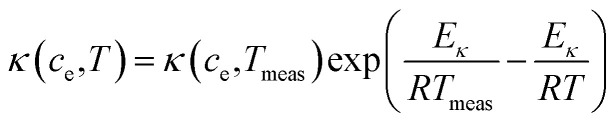
11
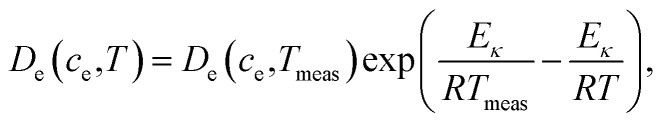
where *E*_*κ*_ is the activation energy for both *κ* and *D*_e_, *T*_meas_ is 298.15 K, *κ*(*c*_e_, *T*_meas_) is a cubic polynomial^56^

12 *κ*(*c*_e_,*T*_meas_) = 1.297 × 10^−10^*c*_e_^3^ − 7.94 × 10^−5^*c*_e_^1.5^ + 3.329 × 10^−3^*c*_e_

and *D*_e_(*c*_e_, *T*) is a quadratic polynomial^56^

13 *D*_e_(*c*_e_, *T*_meas_) = 8.794 × 10^−17^*c*_e_^2^ − 3.972 × 10^−13^*c*_e_ + 4.862 × 10^−10^.

The remaining parameters are taken from Chen *et al.*^56^ and listed in Table 5.

**Table 5:** Other parameters used in the model. All values taken from Chen *et al.*^56^

Symbol	Definition	Value
*c*_eq_	Equilibrium Li^+^ concentration in electrolyte, mol m^−3^	1000
*E*_*κ*_	Activation energy for electrolyte conductivity, J mol^−1^	17 100 (ref. 57)
*F*	Faraday's constant, C mol^−1^	96 485
*Q*_nom_	Nominal capacity, mAh	5000
*R*	Universal gas constant, J K^−1^ mol^−1^	8.314
*t* ^+^	Li^+^ transference number	0.2594
*V*_max_	Upper cutoff voltage, V	4.2
*V*_min_	Lower cutoff voltage, V	2.5
*δ*_s_	Separator thickness, m	1.2 × 10^−5^
*ε*_e_	Separator porosity	0.47

### 3.2 Cell-level degradation mechanisms and their interactions in real-world conditions

The research into the physics of battery degradation has significantly increased as the concern towards cycle life, battery performance, and safety has become the main concern. A comprehensive exploration of lithium-ion battery degradation was conducted in 2005, delving into aging mechanisms occurring at both the cell's anode and cathode electrodes^59^.^34,42,60^ investigated various degradation modes spanning the battery's entire life-cycle and elucidated the cause-and-effect relationships governing these degradation mechanisms. Furthermore,^61^ focused on degradation mechanisms frequently encountered during high-rate charging. Critical interactions between different degradation processes under normal operational conditions, within safety thresholds defined by manufacturers, were examined.^48^ A notable effort to directly correlate diverse degradation mechanisms at the negative electrode is made by.^49^

However, these studies did not specifically investigate degradation patterns and behaviors exhibited by lithium-ion batteries when subjected to real-world driving conditions across varying climates and charging scenarios. Understanding the critical interactions between diverse degradation mechanisms during daily driving cycles is crucial for exploring the impact of each phase of a typical day on battery health. In this work, the three primary degradation modes – loss of lithium inventory (LLI) and loss of active material (LAM) in the positive and negative electrodes^50^ – and their interplay under different operational conditions are discussed.

The overall LLI experienced by a cell, resulting in reduced cyclable lithium, arises from significant side reactions, SEI layer growth, and lithium plating, and an additional, negligible side reaction involves the loss of lithium to the electrolyte.^47,48,62^ The development of the SEI layer immobilizes Li^+^ ions, causing impedance changes due to pore blockage, ultimately leading to LLI.^63^ Lithium plating involves the deposition of lithium ions onto the anode surface, forming a thin layer of lithium metal. This lithium plating creates “dead lithium” by reacting residual lithium with the electrolyte. This interaction forms a high-impedance film, resulting in irreversible lithium loss.^64–66^14
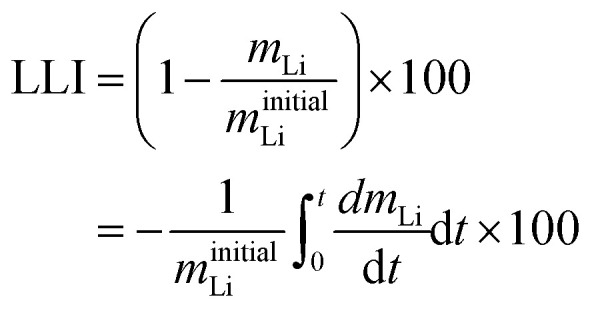
15
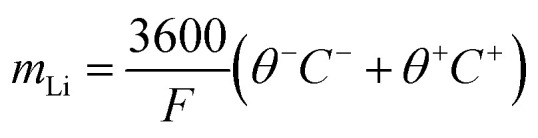


Eqn (14),^50^ defines the overall LLI within a cell, where *m*_Li_ is the total moles of usable lithium in the negative and positive electrode particles, and *θ*^−^ and *θ*^+^ are the scaled volume-averaged particle concentrations of negative and positive electrodes respectively. *C*^−^ and *C*^+^ represent the charge capacity of the negative and positive electrode, respectively.

Due to stresses in the electrode material, Li-ion batteries experience mechanical damage, leading to LAM.^49^ This causes the electrodes to lose their capacity, reducing the available material for electrochemical interactions.^48,50^ The stress in the respective electrodes leads to particle fracture or cracking. The critical modeling equations for LAM due to particle cracking are discussed in the SI Section.16
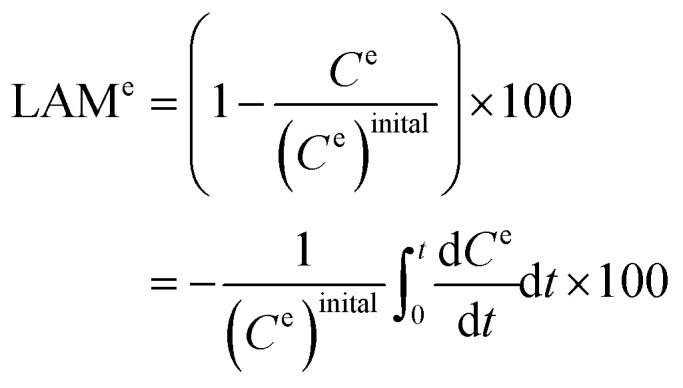


Eqn (16),^50^ defines the LAM within a cell, where the electrode charge capacity, *C*^e^, is defined as17
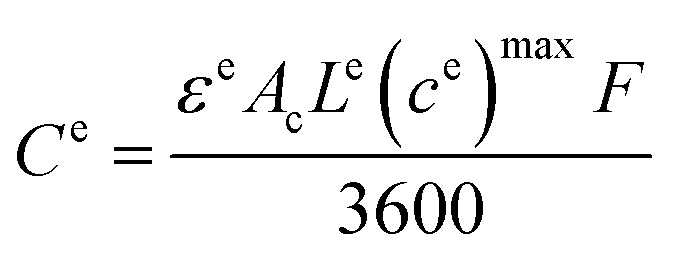
Here, *ε*^e^ is the electrode active material volume fraction, the changes that will cause changes in the electrode capacity. *A*_c_ and *L*^e^ are the surface area of the current collector and electrode thickness, respectively, effectively representing the total electrode volume, and *c*^e^ defines the electrode molar concentration.

Along with the investigation into overall capacity fade, our focus also centers on comprehending the underlying physics of degradation processes in diverse operating conditions involving various daily scenarios and driving patterns across different climatic conditions. A comprehensive discussion of the design of experiments, encompassing key conditions and parameters studied in this work, is presented in the following section.

### 3.3 Calibration of the electrochemical cell model

In this study, an electrochemical model is employed to simulate the behavior of the LGM50 21 700 cylindrical cell. The LGM50 cell contains a graphite-based negative electrode with a 10% SiO_*x*_ mass fraction and a positive electrode made of NMC-811. It utilizes Celgard 2325 as the separator, a tri-layer polypropylene/polyethylene/polypropylene/polyolefin membrane. The electrolyte consists of a 1 mol dm^−3^ LiPF_6_ solution in a mixture of ethylene carbonate (EC) and ethyl methyl carbonate (EMC) in a 3 : 7 volume ratio. The model's parameters are acquired from a comprehensive set of experimental tests conducted by Chen *et al.*^56^ and Nyman *et al.*^67^ These published results extensively characterize the cell's physical, chemical, and electrochemical properties using various experimental techniques. The key parameters and specifications of the LGM50 21 700 cell are presented in the Table 6.^56^ To further validate the electrochemical model, experiments are conducted on an LGM50 21 700 cylindrical cell in a climate-controlled chamber, using a manufacturer-specified cycle life testing method for 50 cycles, and a drive cycle test spanning 7 days is conducted for one of the selected drive cycles, encompassing the entire designed daily cycle pattern.

LGM50 21 700 cylindrical cell parameters and specifications provided by the manufacturer

**Table:** 

Macroscale geometry		
Negative current collector thickness	1.20 × 10^−5^	[m]
Negative electrode thickness	8.52 × 10^−5^	[m]
Separator thickness	1.20 × 10^−5^	[m]
Positive electrode thickness	7.56 × 10^−5^	[m]
Positive current collector thickness	1.60 × 10^−5^	[m]
Electrode height	6.50 × 10^−2^	[m]
Electrode width	1.58	[m]
Cell cooling surface area	5.31 × 10^−3^	[m^2^]
Cell volume	2.42 × 10^−5^	[m^3^]

**Table:** 

Current collector properties		
Negative current collector conductivity	58 411 000	[S m^−1^]
Positive current collector conductivity	36 914 000	[S m^−1^]

**Table:** 

Density		
Negative current collector density	8960	[kg m^−3^]
Positive current collector density	2700	[kg m^−3^]

**Table:** 

Specific heat capacity		
Negative current collector-specific heat capacity	385	[J kg^−1^ K^−1^]
Positive current collector specific heat capacity	897	[J kg^−1^ K^−1^]

**Table:** 

Thermal conductivity		
Negative current collector thermal conductivity	401	[W m^−1^ K^−1^]
Positive current collector thermal conductivity	237	[W m^−1^ K^−1^]

The parameters related to the electrode and cell thermodynamics, kinematics, and transport properties utilized in this work are determined from electrochemical tests performed by Chen *et al.*^56^ on extracted electrode materials. Utilizing a three-electrode configuration with a lithium metal reference electrode allows for determining individual electrode potentials, cell stoichiometry, and lithium content in the positive and negative electrodes. The cell's open circuit voltage (OCV) is also determined based on data obtained from galvanostatic intermittent titration technique (GITT) experiments. The current work employs the physical properties of the cell components, including electrodes, separators, and current collectors, which are characterized through direct measurements after cell tear-down performed by Chen *et al.*^56^ Ion beam milling combined with scanning electron microscopy is used to investigate the pore structures of the positive and negative electrodes and the separator, providing crucial information regarding particle shapes, densities, packing density, and the distribution of conductive carbon and binder domains (CBD).

Furthermore, the chemical and material properties of the cell components utilized in this work are analyzed by Chen *et al.*,^56^ with the elemental composition of SiO_*x*_ and graphite in the negative electrode and NMC composition in the positive electrode determined using energy-dispersive X-ray spectroscopy (EDS) and inductively coupled plasma optical emission spectroscopy (ICP-OES), respectively. Parameters relevant to mass transport phenomena in the electrolyte used in this work are derived from experiments by Nyman *et al.*^67^ to characterize mass transport using various electrochemical methods. Determining ionic conductivity, diffusivity, transport number, diffusion coefficient, and thermodynamic factor of the salt provides essential data for the simulation.

By utilizing such comprehensive experimental data,^56,67^ the simulation results in this work are substantiated, allowing for accelerated cyclic testing experiments while maintaining the validity of the outcomes. This approach saves time and resources, particularly since physically conducting long cycling experiments involving thousands of cycles can be impractical and resource-intensive.

### 3.4 Assumptions

In order to simplify our models and expedite computational processes, we have introduced certain foundational assumptions. These assumptions have been carefully selected to ensure that they do not compromise the integrity of our results. We have outlined these weighted assumptions below:

1. One complex interaction, *i.e.*, between SEI layer growth and particle cracking, which showcases the complex interplay of chemical and mechanical degradation leading to SEI layer growth on the crack, is not considered in this work. However, individually SEI layer growth and particle fatigue happening together are modelled, but the SEI layer's development on fresh cracks is not considered to avoid complexity. The reader is advised to refer to ref. 49 to understand the modeling of SEI on cracks in detail.

2. We have assumed that the loss of lithium in the electrolyte is negligible, and the electrolyte remains stable as electrochemical processes are anticipated to take place primarily within the positive and negative electrodes.^68^ The total lithium concentration in the electrolyte represents less than 2% of the overall lithium content within a cell.^47^ Therefore, neglecting the loss of lithium in the electrolyte does not significantly impact the outcome of our analysis.

3. A key focus of our research involves the phenomenon of SEI layer growth, which we describe using the solvent-diffusion mechanism. Most SEI models found in existing literature are based on the work of Safari *et al.*^63^ According to this body of work, the SEI reaction can be modeled using either the diffusion of solvent molecules towards the graphite surface through the existing SEI (referred to as the diffusion-solvent model) or solvent reduction kinetics at the graphite surface. Empirical evidence suggests that the diffusion-limited model aligns better with experimental data.^49^ Consequently, we have chosen to employ solvent-diffusion-limited SEI growth modeling in our study.

4. Lithium plating on the graphite anode surface is a significant degradation phenomenon among various aging mechanisms. The lithium deposited can be both reversible and irreversible.^62^ Some lithium is deposited on the graphite surface during intercalation through electrical contact. Following a charge transfer reaction with the electrolyte, this deposited lithium will eventually re-integrate into the anode, a process referred to as lithium-stripping.^69,70^ The remaining portion of lithium reacts with the electrolyte to form a high-impedance film, termed “dead lithium”.^65,71^ This loss of lithium is irreversible. Our current research assumes that lithium plating is predominantly reversible, along with dead lithium contributing to the irreversible portion, causing capacity fade and accounting for the loss of lithium due to lithium plating.^72^

5. In this study, a lumped thermal model is considered for simulating the temperature effects of the battery cell, assuming a uniform temperature distribution throughout the cell. The model calculates the volume-averaged cell temperature based on the balance between heat generation within the cell and heat dissipation to the environment. The differential equation governing the temperature evolution incorporates heat generation from electrochemical reactions and ohmic losses, as well as a cooling coefficient that accounts for the cell's geometry and cooling conditions.^73^

## 4 Results

**Fig. 5:**
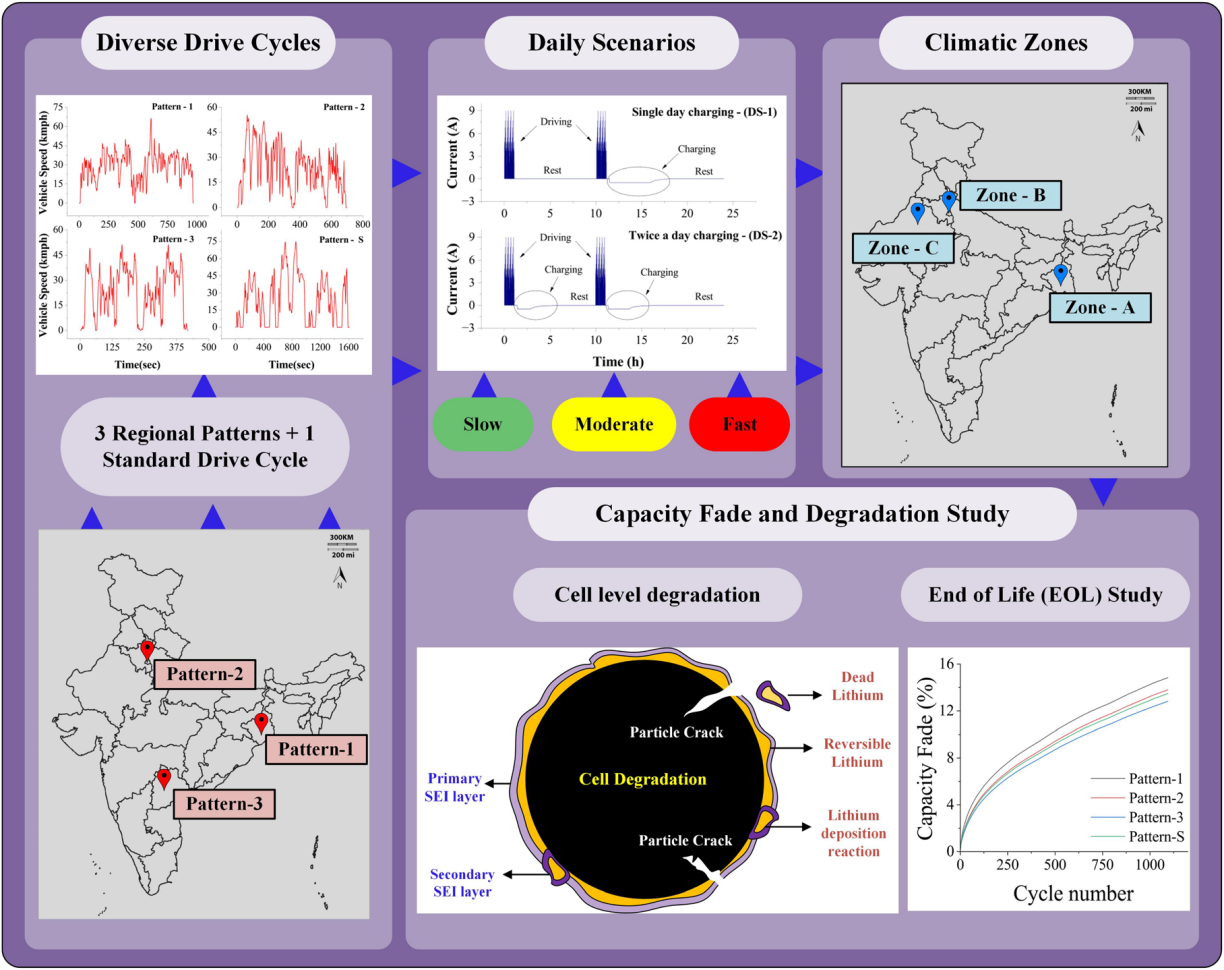
Methodology overview for developing a holistic framework for assessing EV battery end-of-life (EOL).

This section presents the outcomes of the investigation concerning the effects of various parameters, including driving behavior, daily scenarios, climatic conditions, and charge rates, on battery degradation. Fig. 5 depicts the methodology adopted for developing a holistic framework to assess the end-of-life (EOL) of an EV battery, illustrating the key factors and processes considered in this analysis. The analysis explores capacity fade from both chemical and mechanical degradation perspectives, categorizing the capacity fade due to SEI layer growth and lithium plating as chemical degradation, contributing to Loss of Lithium Inventory (LLI) and the mechanical degradation contributed by the fracture of particles within the electrodes due to tensile stress, accounting for the Loss of Active Material (LAM) in both the negative (LAMne) and positive (LAMpe) electrodes. The other pivotal parameters remain constant to discern the specific effects of each analyzed factor in the subsections, allowing for the presentation of focused results. Across the results presented in this section, the cycle number refers to a daily cycle that includes discharge, charge, and rest instances spanning 24 hours, representing one full day. Each cycle number corresponds to a single daily cycle. This study employs simulations on the experimentally verified model of the LG M50 cell to gain insights into battery performance under diverse operational conditions.

### 4.1 Impact of driving behavior on battery aging

**Fig. 6:**
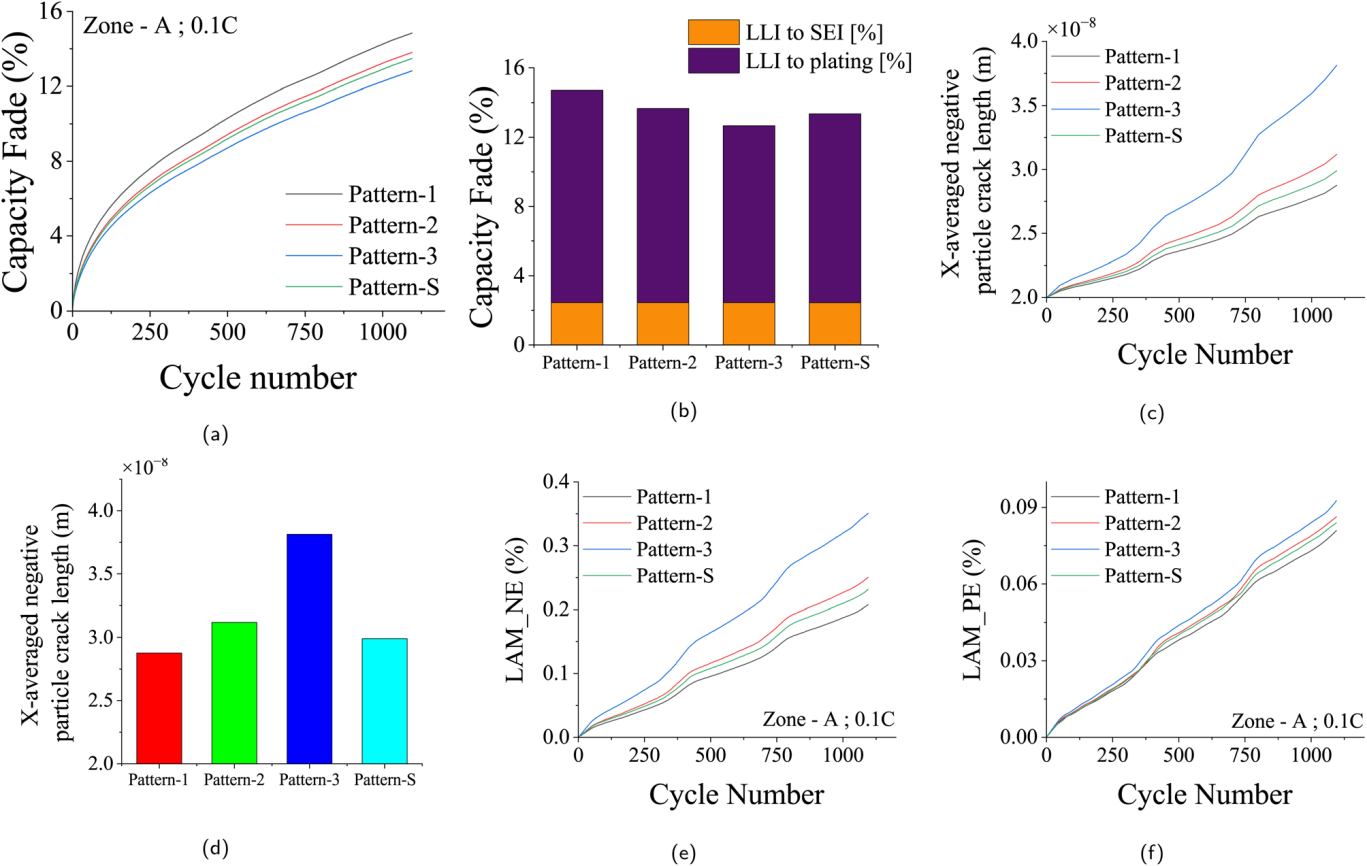
Capacity fade and degradation for different driving behaviors in Zone-A under DS-1 with a 0.1C charge rate. (a) Capacity fade (%) over time (days) for various driving patterns. (b) Contribution of chemical degradation (SEI growth and lithium plating) across patterns. (c) X-averaged negative electrode particle crack growth (*m*) over time. (d) Comparison of particle crack lengths at the 3 year EOL. (e) Loss of active material in the negative electrode (LAMne, %) over time. (f) Loss of active material in the positive electrode (LAMpe, %) over time.

The influence of driving behavior on battery degradation is dissected through a comprehensive analysis of distinct driving patterns: Pattern-1, Pattern-2, Pattern-3, and the standard driving pattern (Pattern-S). A consistent examination is maintained across climatic zones, charging rates, and recharging scenarios, isolating the driving behavior's impact on battery performance. Fig. 6a–f compares driving behaviors for Zone-A climatic conditions, daily scenario-1, and 0.1C charge rate, with each case running for 3 years. Fig. 6a reveals the capacity fade across the 3 years, with Pattern-1 exhibiting the highest fade due to its minimal discharge energy and extended rest period. Individual contributions of chemical degradation phenomena, namely SEI layer growth and Lithium plating, are illustrated in Fig. 6b. SEI layer growth consistently influences varying driving behaviors, while Lithium plating is sensitive to these behaviors. The variation in driving patterns impacts the discharge patterns during which de-intercalation of Li^+^ ions happens at the negative electrode. The tensile stress induced during the de-intercalation leads to LAMne. Fig. 6c–e portray negative electrode particle crack length and LAMne across the driving patterns, highlighting Pattern-3's higher discharge energy and correspondingly increased LAMne. Minimal variation in LAMpe is evident in Fig. 6f, as de-intercalation does not impact the positive electrode during discharging.

The effects of driving behaviors for Zone-A climatic conditions, daily scenario-2, and 0.1C charge rate, reaching a 20% capacity fade before 3 years, are scrutinized in the SI Section (Fig. S1). Fig. S1a depicts the EOL period for different driving patterns. Pattern-3 displays a longer EOL due to its higher discharge energy and reduced rest period. The impact of various driving patterns on chemical degradation, including SEI layer growth and lithium plating, is depicted in Fig. S1b. Similarly, a consistent trend is observed in mechanical degradation behaviors for this scenario. Fig. S1c and d provide insights into the negative particle crack length within the negative electrode and the associated LAMne. Fig. S1e showcases LAMpe across the EOL periods for the various driving patterns.

### 4.2 Influence of daily driving scenarios

**Fig. 7:**
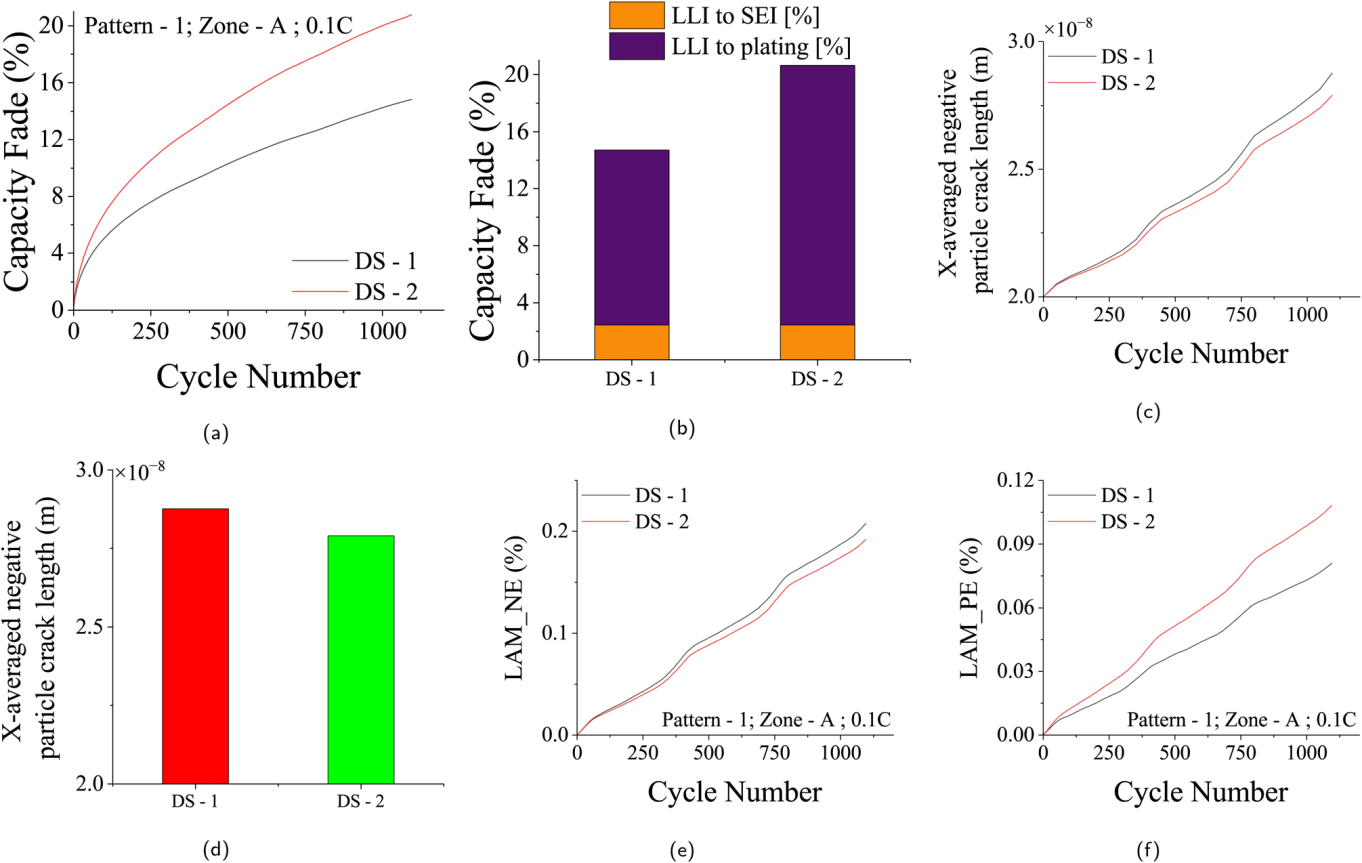
Capacity fade and degradation for two daily scenarios: single daily charge (DS-1) and twice-a-day charge (DS-2) in Zone-A, using driving Pattern-1 and a 0.1C charge rate over 3 years. (a) Capacity fade (%) over time (days). (b) Contribution of chemical degradation (SEI growth and lithium plating) at the 3 year EOL. (c) X-averaged negative electrode particle crack growth (*m*) over time. (d) Comparison of particle crack lengths at EOL. (e) Loss of active material in the negative electrode (LAMne, %) over time. (f) Loss of active material in the positive electrode (LAMpe, %) over time.

This study examines two daily scenarios – single-day charging (DS-1) and twice-a-day charging (DS-2) – to investigate the impact of daily scenarios on aging. By keeping driving patterns, climatic zones, and charge rates constant, the focus remains solely on daily scenario effects. Fig. 7a–f exemplify chemical and mechanical degradation patterns for Pattern-1 driving pattern, considering two daily scenarios DS-1 and DS-2 at Zone-A climatic conditions and 0.1C charge rate. Twice-a-day charging influences capacity fade significantly, as seen in Fig. 7a. The corresponding chemical aging related to capacity fade, including SEI layer growth and lithium plating, is illustrated in Fig. 7b. Increased charging and subsequent de-intercalation introduce higher tensile stress, leading to augmented LAMpe, as shown in Fig. 7f. Notably, while the charging process minimally affects negative electrode tensile stress, Fig. 7c–e display the marginal variations in negative particle crack length and LAMne associated with different driving patterns as de-intercalation doesn't happen at the negative electrode during discharging. The impact of daily scenarios on other driving patterns is similarly depicted in the SI Section (Fig. S2–S4).

### 4.3 Effect of charging rates

**Fig. 8:**
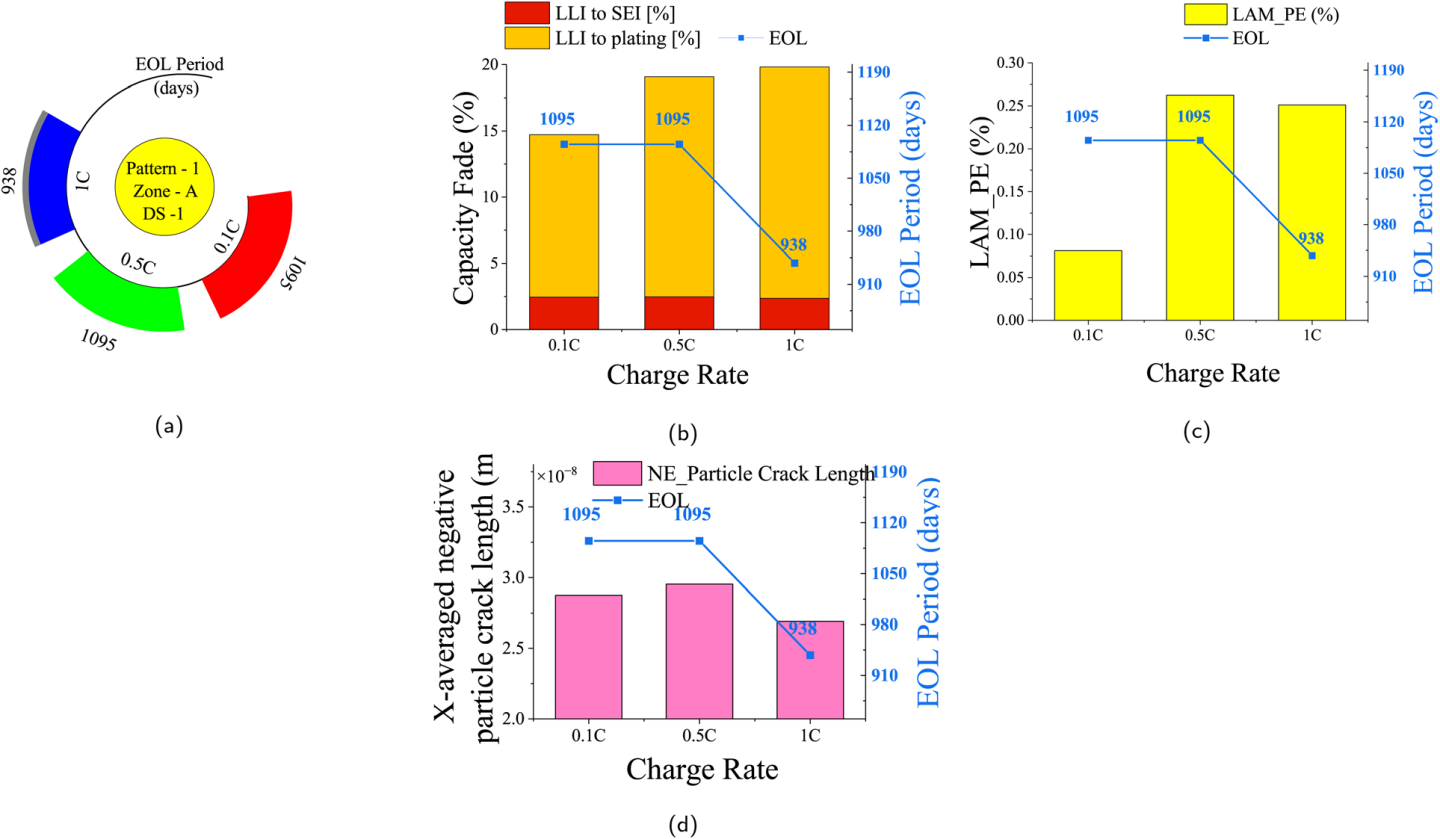
Capacity fade and degradation for three charge rates: 0.1C (slow), 0.5C (moderate), and 1C (fast) under Zone-A climatic conditions, driving Pattern-1, and daily scenario DS-1. Analysis continues until 20% capacity fade or 3 years. (a) EOL periods (days) for each charge rate, with faster fade at 1C. (b) Contribution of chemical degradation (SEI growth and lithium plating) at EOL. (c) Loss of active material in the positive electrode (LAMpe, %) at EOL. (d) Comparison of negative electrode particle crack lengths at EOL.

One critical parameter contributing to capacity fade is the rate at which the batteries are charged. Three different charging rates are considered in this study: 0.1C, 0.5C, and 1C. Fig. 8a–d depicts the capacity fade trends and degradation effects for scenario DS-1, driving Pattern-1 at Zone-A climatic conditions for three different charge rates. The 1C charging rate results in the maximum fading by reaching 20% fade by 938 days, *i.e.*, in 2.56 years, as shown in Fig. 8a. Fig. 8b illustrates the specific contributions of SEI layer growth and lithium plating to capacity fade under different charge rates. As the charging significantly affects the LAMpe, the tensile stress at the PE is high for 0.5C and 1C charge rates, and hence the LAMpe. Fig. 8c shows the impact of 0.5C and 1C charging on LAMpe compared to 0.1C charge rate. However, the particle crack length in the negative electrode (LAMne) remains largely unaffected by the varying charge rates, as shown in Fig. 8d for the same reason that the negative electrode is free from de-intercalation during charging.

In the case of DS-2, the scenario of twice-a-day charging introduces a notable influence on the battery's EOL period. The EOL periods for different charge rates applied to Pattern-1 at Zone-A climatic conditions are presented in the SI Section (Fig. S5a). Charging at a rate of 1C twice a day significantly reduces the battery's lifetime before reaching a 20% capacity fade. The corresponding contributions of SEI layer growth and lithium plating to capacity fade are depicted in Fig. S5b. The increased mechanical stress induced by high charge rates is demonstrated by the impact on LAMpe in Fig. S5c. As no de-intercalation happens, the particle crack length in the negative electrode shows minor variations across different charge rates, as shown in Fig. S5d. The SI Section provides additional insights into the findings for other driving patterns for DS-1 (Fig. S6–S8) and DS-2 (Fig. S9–S11).

A similar analysis is performed by considering different combinations of charge rates within the DS-2 scenario. The morning charge rate is fixed at a particular C-rate, while the evening charge rate is varied for three different rates. Fig. S12 comprehensively illustrates where the morning charge is held at 0.1C, while the evening charge is varied for three charge rates. Fig. S12a provides insights into the capacity fade behavior for Pattern-1 at Zone-A climatic conditions under three different charge rates, showcasing the times before the battery reaches a 20% capacity fade. Fig. S12b depicts the EOL period for different charge rate combinations for the chosen DS-2 scenario. The specific contributions of SEI layer growth and lithium plating to capacity fade are depicted in Fig. S12c.

Importantly, the mechanical stress experienced by varying charge rates gives rise to distinct EOL periods, resulting in significant variations in LAMpe, as observed in Fig. S12d. The 1C charge rate yields 0.12% LAMpe by its EOL of 597 days, while the 0.5C charge rate achieves 0.19% LAMpe by its EOL period of 785 days. In contrast, the 0.1C-rate results in merely 0.09% LAMpe even after 999 days. However, varying charge rate has minimal impact on particle crack length at the negative electrode and correspondingly on LAMne as shown in Fig. S12e, as de-intercalation does not occur at the negative electrode during the charging phase. The SI Section expands upon these findings for the scenarios of fixed 0.5C and 1C morning charges (Fig. S13 and S14).

### 4.4 Across different climatic conditions

**Fig. 9:**
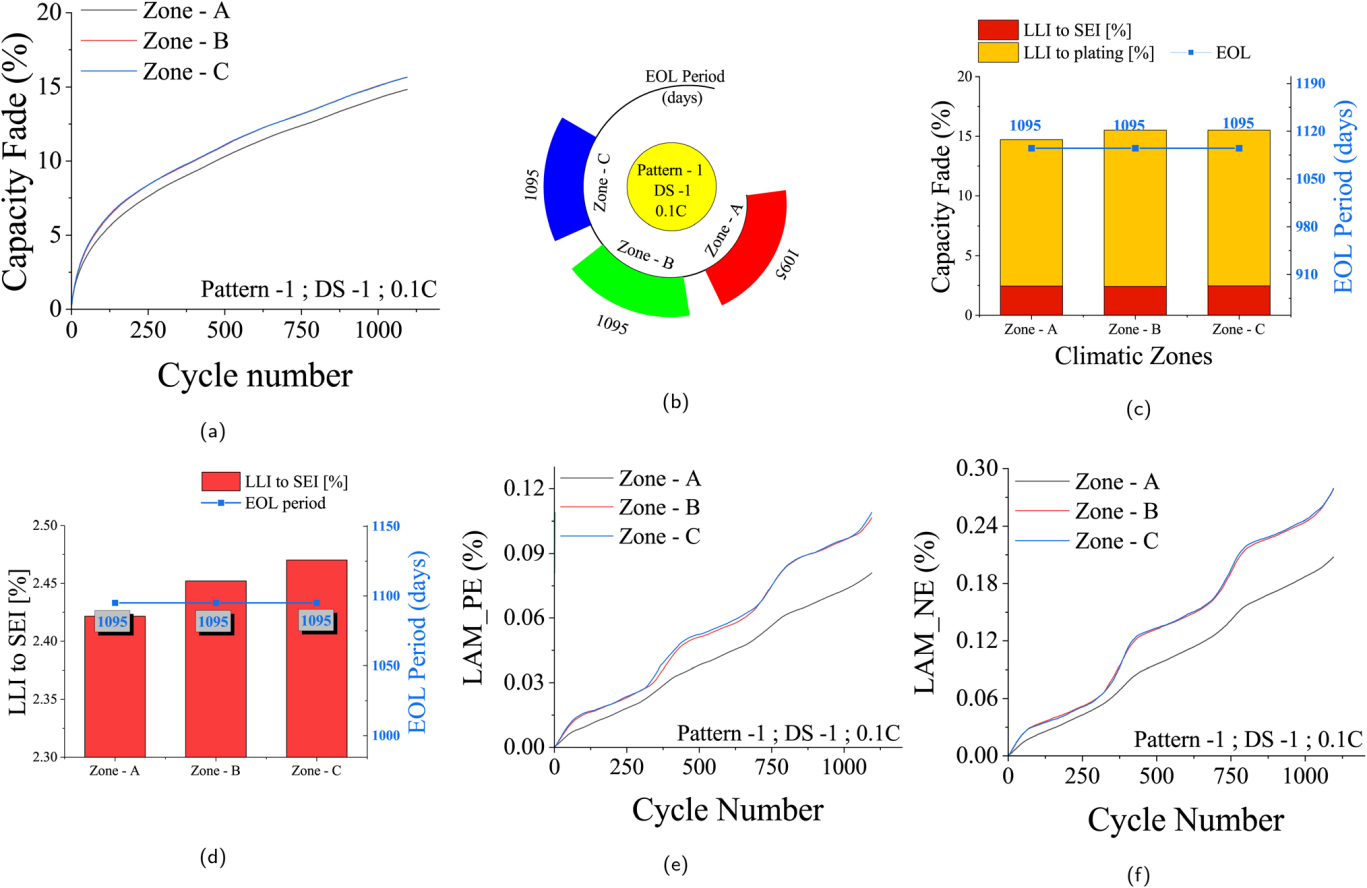
Capacity fade and degradation characteristics for three climatic zones (Zone-A, Zone-B, Zone-C) under driving Pattern-1 and daily scenario DS-1 with a 0.1C charge rate over 3 years. (a) Capacity fade (%) over time (days). (b) EOL period (days) showing time to reach EOL criterion; all zones reach EOL in 3 years. (c) Contribution of chemical degradation (SEI growth and lithium plating) at EOL. (d) LLI due to SEI layer growth (%) at EOL, highlighting temperature dependence with higher SEI growth in Zone-C. (e) Loss of active material in the positive electrode (LAMpe, %) over time. (f) Loss of active material in the negative electrode (LAMne, %) over time.

The impact of temperature and climatic conditions on battery degradation is pivotal. Extreme temperatures can significantly aggravate both chemical and mechanical degradation processes. This study delves into the impact of varying climatic conditions – Zone-A, Zone-B, and Zone-C – on battery aging. Zone-A, situated in the eastern region with yearly temperature extremes of 12 °C and 41 °C, demonstrates minimal fading. On the other hand, Zone-B and Zone-C, representing Delhi and Churu respectively, exhibit more substantial degradation due to extreme yearly temperatures of 5.6 °C to 45.4 °C and 1.8 °C to 47.2 °C, respectively. Notably, Zone-B and Zone-C showcase similar temperature profiles, leading to comparable impacts on degradation. For the Pattern-1 and 0.1C charge rate under the DS-1 scenario, the effects of varying climatic zones are evident in Fig. 9a–f.

As depicted in Fig. 9a, Zone-B and Zone-C, with similar temperature characteristics, yield almost identical loss of lithium inventory by the end of the standard 3 year EOL period. Conversely, Zone-A experiences less fading due to its milder temperature ranges. The corresponding EOL periods are as shown in Fig. 9b. Fig. 9c depicts the dependency of chemical degradations – SEI layer growth and lithium plating – on climatic conditions. From Fig. 9d, the temperature dependency of SEI layer growth is evident, where a greater rate of SEI layer growth is observed under Zone-C climatic conditions. As temperature effects influence both positive and negative electrodes, increased reaction kinetics at higher temperatures amplify stress in both electrodes. This results in noticeable variations in LAMpe and LAMne under different temperature conditions, as shown in Fig. 9e and f. Further details are available in the SI Section for Pattern-2, Pattern-3, and Pattern-S driving patterns, using a 0.1C charge rate and the DS-1 scenario, across the three climatic zones (Fig. S15–S17).

The capacity fade behavior and respective EOL periods across three zones for Pattern-1, a 0.1C charge rate, and the DS-2 scenario are illustrated in the SI Section (Fig. S18a and b). Zone-A offers an EOL of 999 days, while Zone-B and Zone-C share a similar extended EOL period of 915 days and 900 days, respectively, providing less longevity before a 20% capacity fade occurs. The influence of temperature is further highlighted through the SEI layer growth and Lithium plating contributions to overall fading, as shown in Fig. S18c. The temperature-dependence of SEI growth is evident in Fig. S18d, with Zone-C displaying a 2.33% fade by its EOL of 900 days, while Zone-A exhibits slightly less degradation than Zone-B and Zone-C even after an extended EOL period of 999 days. The mechanical damage incurred at the positive and negative electrodes due to varying climatic conditions is visually presented in Fig. S18e and f. These insights are further detailed in the SI Section for Pattern-2, Pattern-3, and Pattern-S driving patterns, using the DS-2 scenario, with a 0.1C charge rate, across the three climatic zones (Fig. S19–S21).

Similarly, Fig. S22 assesses the impact of climatic variation on battery degradation for Pattern-1, a fixed 0.1C morning charge rate, and a 0.5C evening charge rate. Zone-B and Zone-C demonstrate an accelerated EOL of 20% capacity fade at 630 and 616 days, respectively, as shown in Fig. S22a and b. In contrast, Zone-A allows for a more prolonged EOL period, reaching 785 days due to its milder yearly temperature extremes. Fig. S22c portrays the associated chemical degradation effects, and the impact of temperature, specifically on SEI layer growth, is further highlighted in Fig. S22d, while Fig. S22e and f present the consequential LAMpe and LAMne, showcasing the differences between Zone-A, Zone-B, and Zone-C. In conclusion, the presented results underscore the intricate interplay of parameters on battery degradation, revealing the multifaceted nature of this phenomenon across diverse operational conditions.

### 4.5 Experimental validation of the electrochemical cell model

**Fig. 10:**
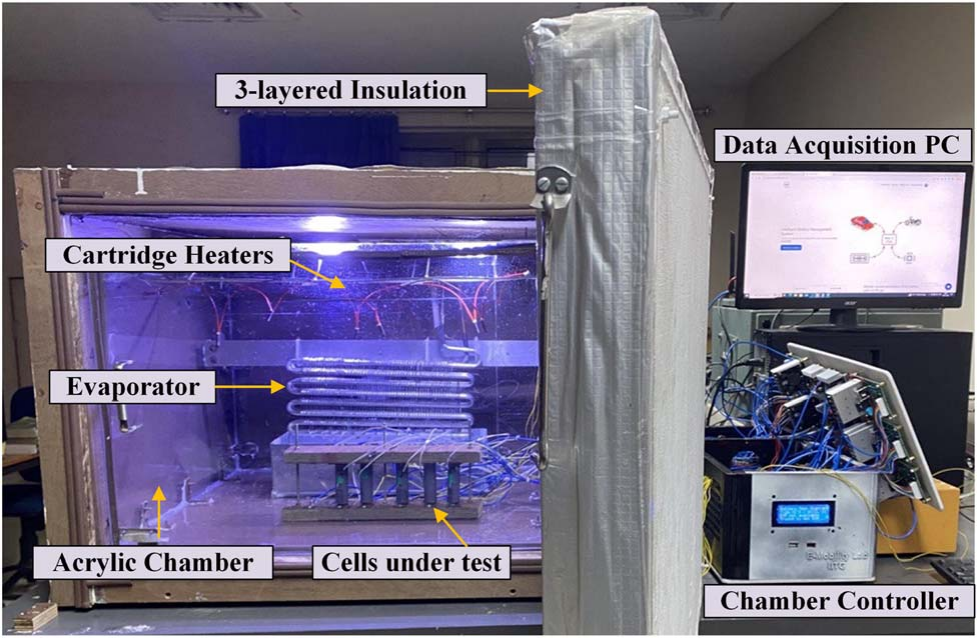
Indigenously developed test chamber with integrated heating/cooling units. The insulated acrylic chamber is operated by a chamber controller that manages data acquisition and sends results to a local desktop for visualization and post-processing.

The electrochemical model employed in this study has undergone rigorous validation through extensive experimental work. To enhance the credibility of the simulation results, two different experiments – (1) The manufacturer specified a standard cycle life experiment and (2). Drive cycle experiments were conducted on the selected LGM50 cell within our laboratory. For this purpose, an environmentally controlled battery test chamber (Fig. 10) was developed in-house, providing precise temperature control with an accuracy of ±1 °C and robust temperature leakage protection.^74^

The chamber, constructed primarily from acrylic, features a front-loading design with a hinged lid and an internal volume capacity of 175 liters. Ceramic cartridge heaters, totaling eight and operating on a 12 V DC supply each, ensure above-room temperatures are reached evenly throughout the chamber. A refrigeration unit based on fundamental thermodynamic heat exchange processes regulates the chamber's temperature below room temperature, aided by a strategically placed DC cooling fan. The chamber accommodates six cells simultaneously with a six-channel supported battery holder. An advanced three-layered (plywood-glass wool-plywood) thermal insulation housing, coated with heat-reflective paint, maintains a constant internal temperature while preventing heat exchange with the surroundings.

The test bed controller, featuring the central microcontroller unit (Arduino Mega2560 pro), serves as the central control unit for the chamber. The microcontroller oversees operations such as data acquisition, charge–discharge control, and temperature regulation. Utilizing a comprehensive data acquisition unit equipped with sensors, including a 4-wire voltage measurement setup, ACS712 current sensor modules, DHT22 digital sensor, and DS18B20 waterproof temperature probe, the chamber accurately measures cell parameters, chamber humidity, and temperature. Furthermore, a charge–discharge controller unit, comprising 300 W, 20 A-rated step-down DC–DC buck converter modules for individual cell charging and programmable electronic loads with MOSFETs for efficient cell discharging, ensures precise control over battery currents. The chamber temperature controller unit, leveraging the DS18B20 temperature probe and electro-mechanical relays, maintains uniform temperature distribution within the chamber.

In conducting experiments, addressing experimental uncertainty is critical to ensure the reliability and accuracy of the results. This uncertainty primarily stems from sensor measurements, which must be meticulously calibrated to ensure precision. Each sensor within the testing chamber and charge–discharge controller must undergo rigorous calibration against known values before integration, enhancing measurement accuracy. However, despite calibration efforts, potential issues may arise during experiments, such as the release of flammable gases during high-temperature testing, posing risks of thermal runaway and chamber explosions. To mitigate these risks, high-temperature-tolerant materials need to be utilized for chamber construction, and specific gas sensors should be incorporated for safety monitoring. Additionally, measures need to be taken to prevent short circuits, chamber leakage, and temperature instability through proper insulation and sealing techniques. Addressing these uncertainties not only enhances the credibility of the experiment results but also ensures the safety and reliability of the experimental setup.

#### 4.5.1 Manufacturer specified standard cycle life experimentation

A manufacturer-specified standard cycle life experiment in the chamber for 50 cycles. Another experiment subjected the chosen cell to one of the daily driving cycle tests for 7 days at different ambient temperatures. The results obtained from the actual experimentation were then compared with the simulation tests by replicating the same two experiments in the simulation environment using the electrochemical model coupled with the ageing model by integrating degradation effects, ensuring their agreement.

**Fig. 11:**
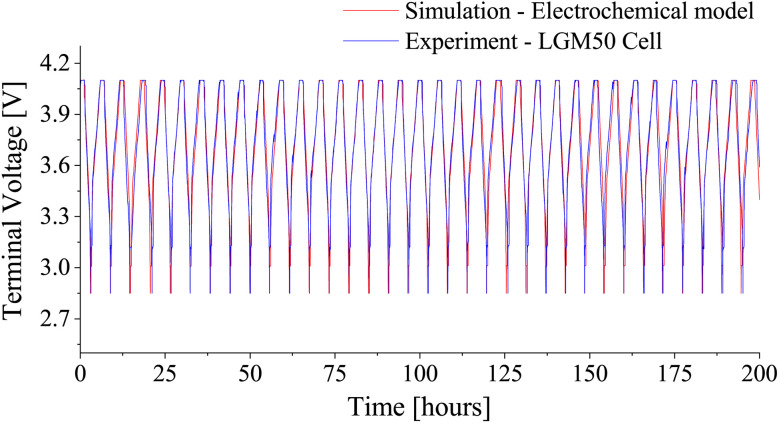
Comparison of terminal voltage profiles for LGM50 during cycle life testing. 200 hour voltage profiles: simulation *vs.* experiment.

In the manufacturer-specified cycle life experiment, the LGM50 cell underwent a Constant Current Constant Voltage (CCCV) charging process involving a constant current rate of 0.3C (1.44 A) until the cell voltage reached 4.1 V, followed by constant voltage charging at 4.1 V until the current reached 240 mA. Subsequently, a rest period of 10 minutes was introduced, and the cell was discharged at a constant current rate of 0.5C (2.4 A) until the voltage reached 2.85 V, followed by a rest period of 20 minutes. This cycle was repeated for 50 cycles at an ambient temperature of 25 °C. Comparing the terminal voltage profiles obtained from experimental and simulation data over 200 hours (Fig. 11), the two profiles displayed a close match, and the analysis of total energy exchange during the cycle life experiment further verified the accuracy, as the experimental and simulation values exhibited a small difference of close to 0.46%.

#### 4.5.2 Drive cycle experimentation

**Fig. 12:**
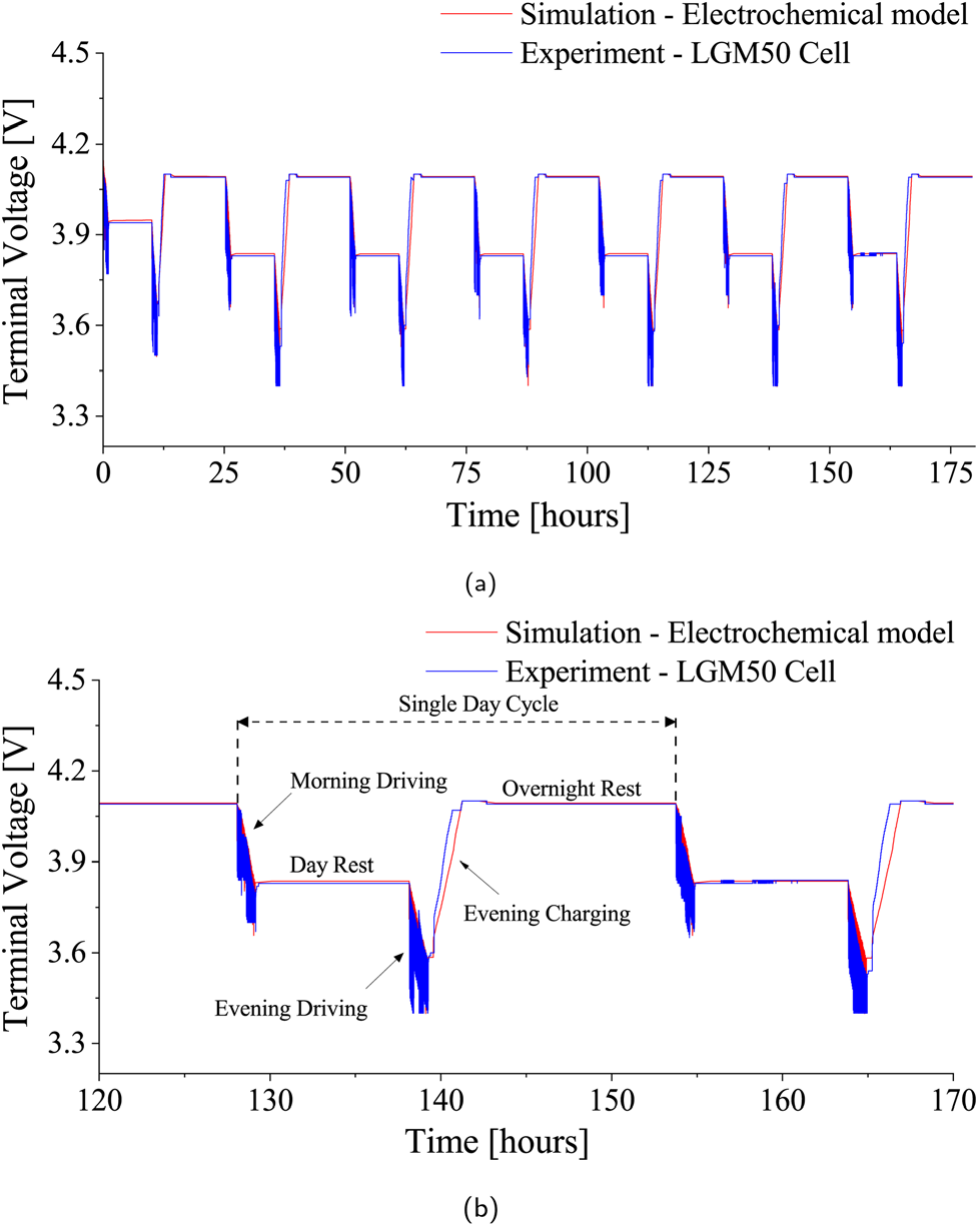
Comparison of terminal voltage profiles for LGM50 under drive cycle conditions at 25 °C ambient temperature. (a) Week-long voltage profiles: simulation *vs.* experiment. (b) Daily cycle voltage profiles: simulation *vs.* experiment.

**Fig. 13:**
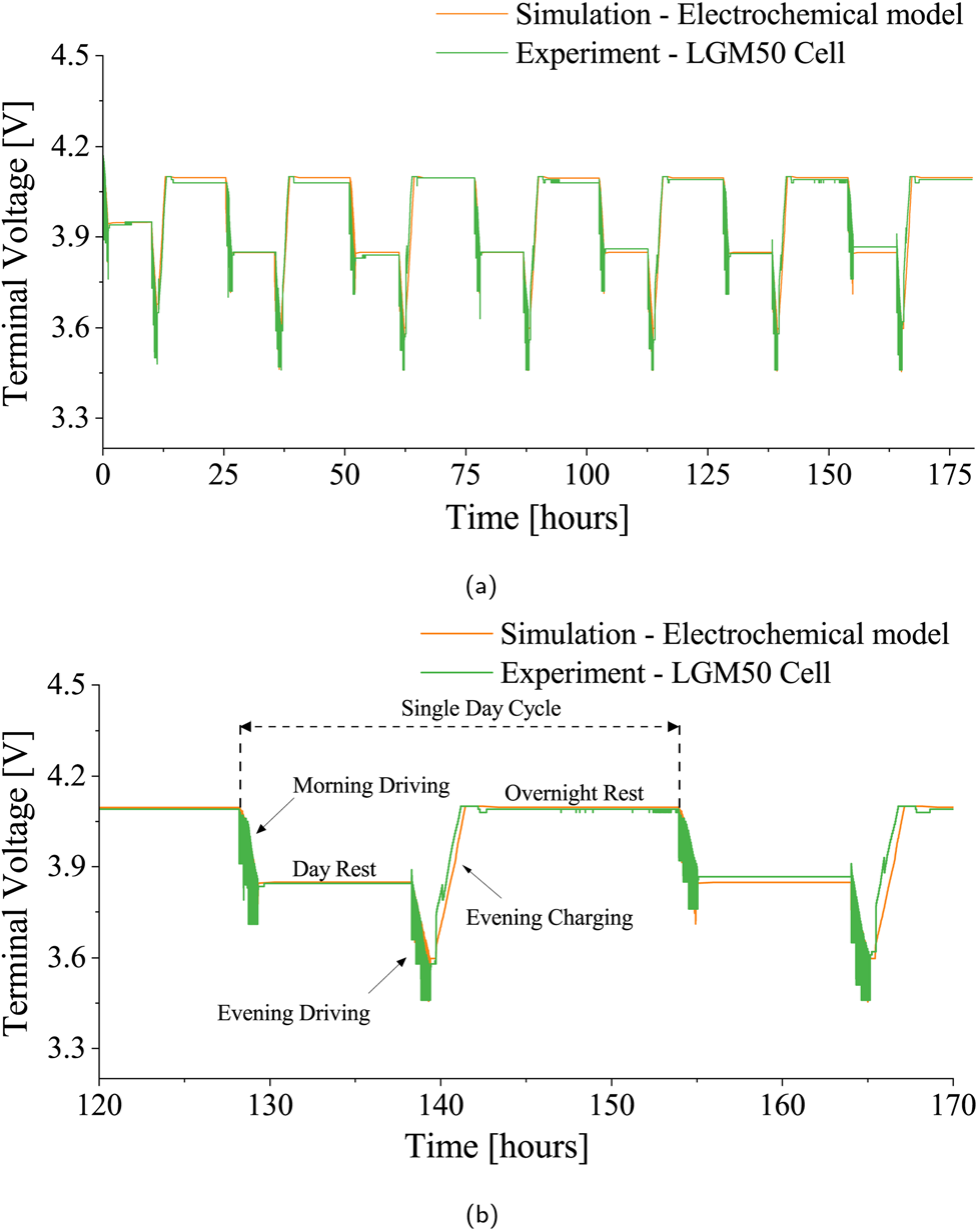
Comparison of terminal voltage profiles for LGM50 under drive cycle conditions at 40 °C ambient temperature. (a) Week-long voltage profiles: simulation *vs.* experiment. (b) Daily cycle voltage profiles: simulation *vs.* experiment.

Another drive cycle test was conducted for one of the chosen patterns (Pattern-1) on the LGM50 cell, simulating the complete daily cycle an EV would experience, including charging and rest stages within a daily scenario-1 (Fig. 3e). The LGM50 cell underwent this daily pattern for 7 days at two different ambient temperatures, 25 °C and 40 °C, respectively, and the results were compared with the simulation data. Fig. 12a and 13a illustrate closely matching voltage profiles from experimental and simulated ambient temperature data. Additionally, the comparison of total energy exchange values for both experimental and simulation cases, which were 150.85 Wh and 151.97 Wh at 25 °C and 153.55 Wh and 153.30 Wh at 40 °C, respectively, with an error of less than 0.74% at 25 °C and 0.16% at 40 °C, demonstrated further accuracy. These results establish the accuracy and reliability of the electrochemical model in replicating the behavior of the LGM50 cell under various test conditions. Using an accurate and comprehensive parameter set obtained through thorough experimental work by Chen *et al.*^56^ and Nyman *et al.*,^67^ coupled with the experimental results from our laboratory, significantly strengthens the validity of the chosen electrochemical model. Furthermore, it is possible to fine-tune the parameters obtained from Chen *et al.*^56^ and Nyman *et al.*^67^ when integrating them into the electrochemical model, based on the experimental results obtained.

## 5 Discussion

In contrast to conventional belief, the analysis uncovered a rather fascinating trend in battery performance concerning various drive cycle metrics: KI, RPA, and PKE. Although it's generally assumed that drive cycles with higher KI, RPA, and PKE would contribute to more significant battery fading, the actual pattern of fading observed was quite intriguing: Pattern-3 < Pattern-S < Pattern-2 < Pattern-1. This deviation from expectations led us to look closely at the underlying factors contributing to battery degradation. On a comprehensive analysis of battery usage over a complete day's drive cycle, it is found that relying solely on the measurements of drive cycle metrics no longer directly indicates battery fading. Instead, the interplay of various factors, such as driving duration, charging intervals, and resting periods, plays a crucial role in determining the overall extent of fading.

Further investigation led to the realization that the average speed during a drive cycle and the drive cycle metrics are pivotal in determining fading. A drive cycle with high KI but lower average speed would result in more discharge energy being expended to cover a prescribed daily driving distance, as it takes longer to complete the same distance. This extended time frame for completing the distance results in a shorter resting period during the day. This resting period's length is a key factor in battery degradation, as more extended periods of rest lead to increased calendar aging of the battery.

Interestingly, when considering the discharge energies for the different drive patterns in this study, they followed this order: Pattern-1 < Pattern-2 < Pattern-S < Pattern-3. This means that Pattern-3, with its lower rest period, experiences less capacity fade. On the contrary, Pattern-1, with lower drive metrics and higher average speeds, results in lower discharge energies per day, leading to longer rest periods and, ultimately, higher overall fading. Given a constant charging rate, the complex interplay of discharge energy, drive cycle metrics, average speed, and rest periods gives rise to intricate dynamics governing battery degradation and capacity fade.

Moreover, our study revealed that extended charging cycles and higher charging rates lead to notable reductions in battery capacity due to a loss in usable energy. Rapid charging accelerates degradation, causing both capacity and power fade. Elevated temperatures during charging contribute to accelerated battery aging. Longer charging times amplify temperature rise and further aggravate fading. In unison, variations in key parameters – driving behavior, charging scenarios, charging rates, and temperature – cumulatively contribute to the loss of lithium inventory and, consequently, overall capacity fade.

Our investigation in the realm of battery degradation unveiled that the chemical processes play a pivotal role, emerging as a major reason for the overall capacity fade. A critical phenomenon within this is lithium plating, a key chemical degradation process that needs careful attention. Notably, lithium plating on anode, also known as anode plating, is significantly influenced by charging times and rates.^75–78^ Longer charging durations facilitate increased intercalation of Li+ ions into the anode, raising the likelihood of Li^+^ ions accumulating on the anode's surface, thereby intensifying lithium plating. Furthermore, charging at elevated rates, particularly in CCCV charging protocols, triggers lithium plating due to the prolonged CV phase.^72^ At high charging rates, more Li^+^ ions get deposited on the electrode/electrolyte interphase as the Li^+^ ions diffusion coefficient is much higher than the diffusion coefficient of solid lithium. This results in a high concentration gradient accumulation of Li^+^ ions at the anode interface. The saturation of these Li ion concentrations leads to anode plating.^75^

On the other hand, another critical chemical degradation process involves the growth of the Solid Electrolyte Interphase (SEI) layer, which progressively thickens over cycles and usage. Our analysis determined that the capacity loss attributed to SEI layer growth is primarily time-dependent and remains consistent across various charge rates, charging durations, discharge patterns, and cyclic conditions. Factors such as the total cycle time or the duration under scrutiny (as in this study, a span of 3 years or the period leading to a 20% capacity fade) and operating temperature are the critical factors that account for the SEI layer formation.^79^ Higher temperatures accelerate the formation rate of the SEI layer.^34,80^ In totality, while capacity loss due to stable SEI layer formation is minimal, the dominant contributor to chemical degradation is the rapid capacity loss incurred by lithium plating at the electrode surface.^79,81,82^

An equally significant phenomenon within the battery degradation realm is mechanical deformation and fatigue, which a battery would undergo during daily operations. Electrode particles within the battery experience mechanical stress, leading to reduced capacity, resulting in LAM. Particle cracking resulting in LAM is considered in this work and is critically analysed for different operating conditions.^49^ During the intricate process of Li^+^ ion de-intercalation – occurring at the negative electrode during discharge and at the positive electrode during charge – the respective electrodes experience positive tensile stress. Notably, LAM is solely influenced by tensile stress during de-intercalation.^49^ Longer charging times and higher charging rates increase stress on the electrodes, significantly leading to LAM.^42,48^ It is found that the interplay between varying charge rates and the daily charging scenarios, including both single and twice-a-day charging patterns, intensifies the mechanical stress experienced by the positive electrode.

A direct correlation emerges between drive cycle metrics and mechanical degradation. Increased KI, as well as higher values of RPA and PKE, are associated with greater mechanical degradation. Furthermore, our study determined that diverse discharge patterns, shown by distinct drive cycles, introduce increased stress to the negative electrode. Moreover, the negative electrode critical stress factor is lower than that of the positive electrode. Hence, the positive electrode can be subjected to higher tensile stress conditions, resulting in a proportionately less occurrence of LAM.^49^ It's important to note that harsher driving behavior leads to elevated mechanical degradation. However, it doesn't directly contribute to overall fading, encompassing both chemical and mechanical degradation. Chemical degradation, unlike mechanical degradation, depends not only on drive cycle metrics but also on rest conditions. Mechanical degradation also depends on the operating temperature conditions of the battery. In extreme temperatures, specifically at lower temperatures, the dominant path of battery degradation is particle fracture.^49^ Both negative and positive electrodes are affected by the temperature, and it is evident from our results that both electrodes show a variation when subjected to different climatic conditions.

This research offers a comprehensive framework to evaluate the battery end-of-life (EOL) for electric vehicles, unveiling the complex interactions between different daily driving behaviors, varied charge rates and frequencies, and rest periods across varied climatic conditions, and their combined effect on battery degradation. The findings challenge the traditional assumptions about battery capacity fade, revealing that high kinetic intensity (KI) drive cycles don't always lead to quicker fade. Instead, the collective impact of average speed, charging frequency, rest duration, and the climate at which the vehicle is operating appears to be more decisive in battery longevity. Surprisingly, patterns with lower kinetic intensity and higher average speeds were found to experience more significant degradation due to prolonged rest periods that exacerbate calendar aging. For instance, although a drive cycle with a high KI might suggest intense battery use, if such a cycle involves lower average speeds, it could lead to more energy used over a longer period, reducing the rest time for the battery and consequently, lessening calendar aging. This unexpected trend suggests that it’s not just the driving metrics that matter but a combination of factors, including the length of driving, frequency of charging, duration of resting periods, and the environmental conditions that shape a battery's lifespan.

Furthermore, the study underscores the detrimental effects of rapid charging and elevated temperatures on battery capacity. Fast charging rates accelerate the onset of lithium plating, contributing to substantial capacity fade, while high temperatures during charge cycles exacerbate the aging process, with the notable processes of lithium plating and SEI layer growth playing central roles in capacity fade. Mechanical factors, particularly the tensile stress exerted on electrode particles during charge cycles, are also highlighted as a significant degradation pathway. The study's analysis suggests that more prolonged charging at higher rates induces greater mechanical stress, particularly on the positive electrode, which in turn increases the likelihood of capacity loss due to mechanical strain.

**Fig. 14:**
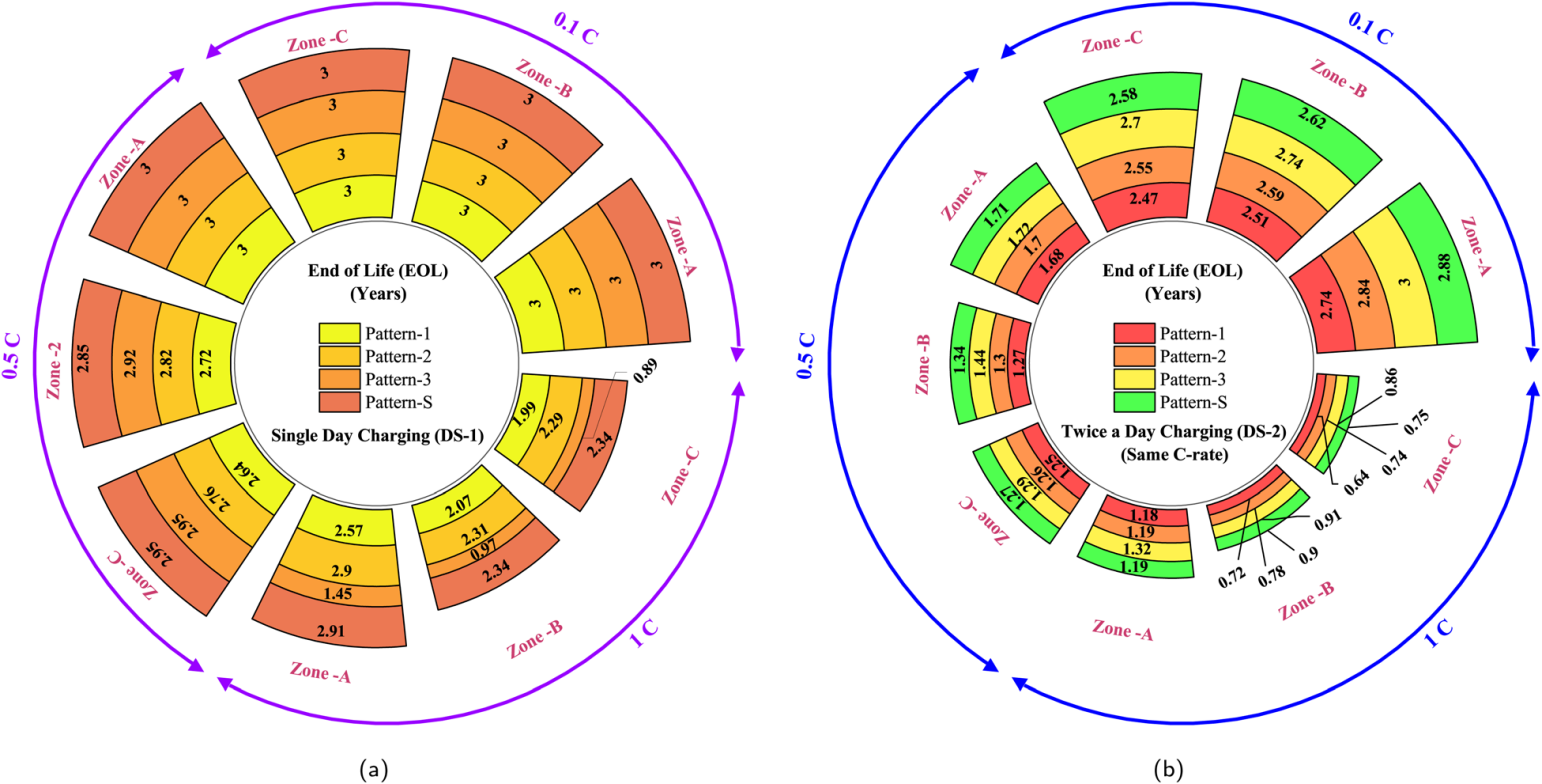
Summary of EOL periods (in years) for all chosen driving patterns across three climatic zones, for three charge rates (a) within daily scenario-1 (b) within daily-scenario-2.

**Fig. 15:**
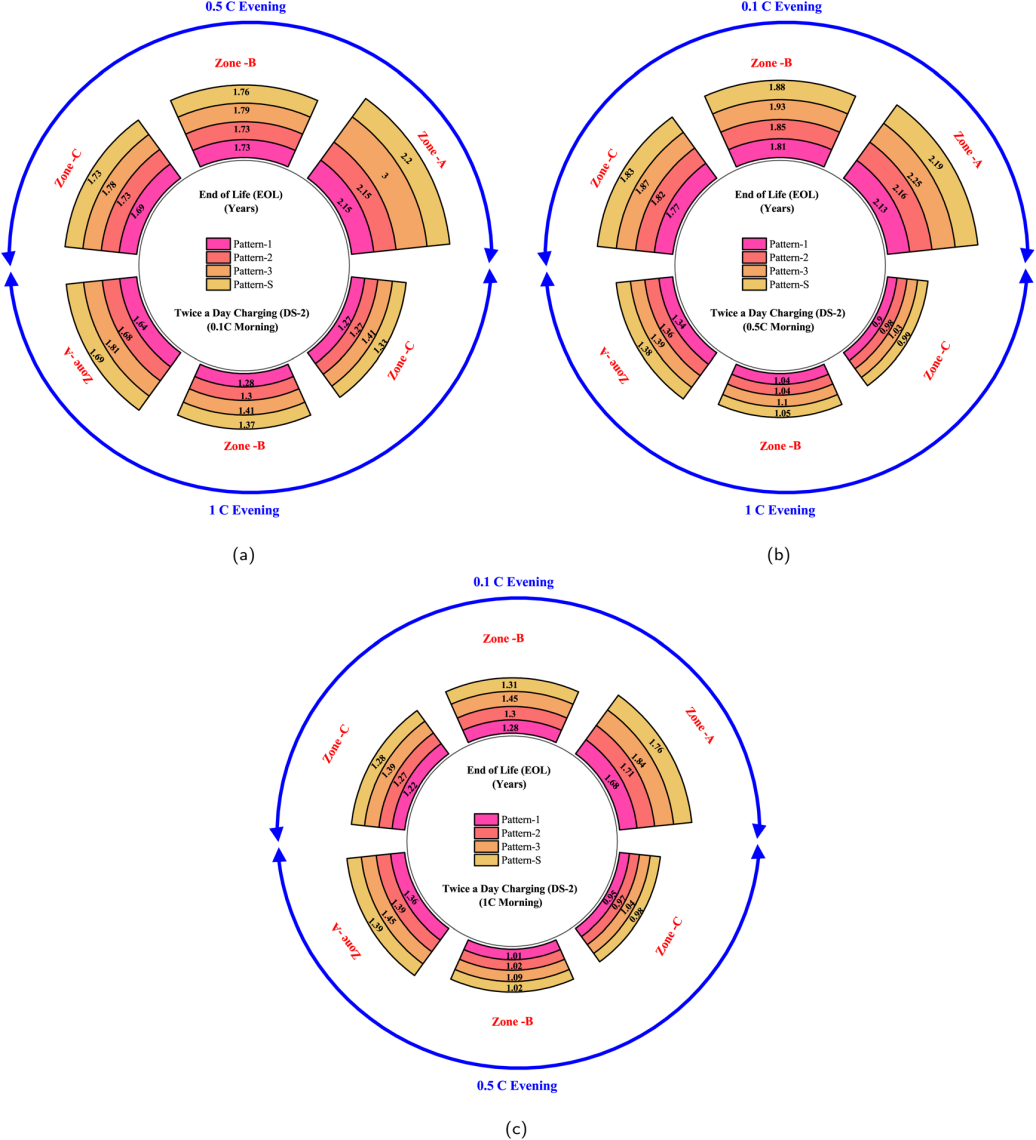
Summary of EOL periods (in years) for all chosen driving patterns across three climatic zones, and for three evening charge rates within daily scenario-2 (a) at a constant morning charge rate of 0.1C (b) at a constant morning charge rate of 0.5C, (c) at a constant morning charge rate of 1C.

The combined effect of all these factors together, along with their examined EOL periods, is integrated and presented in Fig. 14. Fig. 14a illustrates the EOL periods for DS-1, encompassing all four driving patterns, varying charge rates, and climatic zones. Additionally, Fig. 14b displays the EOL periods for DS-2, involving twice-a-day charging with consistent morning and evening charge rates. Meanwhile, Fig. 15a–c detail the EOL periods for DS-2, maintaining a fixed morning charge rate while varying the evening charge rate. This study proposes an EOL assessment framework for two-wheelers, utilizing the LGM50 21 700 cylindrical cell electrochemical model, which has a nominal voltage of 3.6 V and a nominal capacity of 5 Ah as the reference battery. The LGM50 cell is commonly used in two-wheeler battery packs, making it an appropriate choice for this study. It should be noted that while the obtained EOL model values may vary with different cell chemistries and geometries, the insights developed and the underlying processes remain consistent, irrespective of the cell geometry and chemistry. The findings indicate that driving patterns characterized by higher average speeds, longer rest periods, frequent charging sessions throughout the day, and exposure to elevated temperatures lead to accelerated battery fading compared to other operating conditions.

In comparison with existing studies, this work advances the state-of-the-art by providing a more comprehensive and realistic end-of-life (EOL) evaluation framework for electric vehicle batteries. For example,^33^ quantified capacity loss for different daily driving scenarios using real-world data, observing a maximum capacity loss of 14.9% after 100 000 miles, but did not map the complete degradation trajectory to EOL. Similarly,^25^ examined the effect of driving styles and traffic conditions using 240 cycles of real highway data and reported a maximum capacity fade of only 0.5%, again without extending to full EOL.^15^ developed a combined calendar and cycle aging model with a lifetime estimate of 150 000 cycles for elevators and vehicle V2G use but did not consider realistic two-wheeler driving patterns, diverse climates, or varying charging practices.^16^ assessed combined driving and V2G effects, testing cells for up to 2400 cycles and then predicting EOL at around 5300 cycles (at 80% SOH) using linear regression, without validating under region-specific or realistic daily variations. Notably,^18^ studied impacts of driving cycles, ambient temperatures (0 °C, 20 °C, 40 °C), charging modes, and trip distances on EOL, showing that low temperatures can reduce battery life by up to 8.6 times compared to optimal conditions, and fast charging can vary EOL by ±30% depending on ambient temperature. Yet their driving scenarios relied on standard cycles and fixed conditions rather than realistic, dynamic profiles. In contrast, this work uniquely combines diverse driving styles, customized regional drive cycles, realistic rest and charging behaviors, and varying climatic conditions into a unified framework, tracking the full capacity fade to EOL. This detailed, real-world mapping fills a critical research gap, providing actionable insights for OEMs, researchers, and policymakers to improve region-specific battery design, usage strategies, and lifetime prediction. However, the calibration and experimental validation in this study rely on fresh cells, but the findings can be extended to include validation considering degradation effects. Although calibrating the degradation effect is both challenging and costly, if resources and conditions allow, validating the findings with degradation effects and the aging process would further enhance the reliability and strengthen the study.

## 6 Limitations of the study

This study uses three representative driving patterns from rural, urban, and high-traffic stop-and-go scenarios in India to highlight realistic battery usage conditions. While these patterns offer valuable insights, they do not capture the effects of driving in mountainous terrains, and road gradients were not explicitly modeled. Slope-related forces, which can significantly influence traction effort, power demand, and overall battery performance, were discussed in the motivation section and detailed in prior reports. However, the proposed framework is designed to be flexible and can easily be extended to include region-specific driving cycles with gradients and other unique conditions.

Similarly, the study assumes a Constant Current–Constant Voltage (CCCV) charging protocol, which reflects a common and practical approach but does not account for real-world charging interruptions that can further impact battery degradation. The framework remains modular and can incorporate other charging strategies, such as pulse or interrupted charging, as well as additional climatic profiles and usage scenarios. While the specific EOL figures reported may vary with different conditions, the methodology provides a robust and adaptable approach that can be refined to support more comprehensive battery End-of-Life assessments in future work.

## 7 Conclusion and future scope

Based on the current findings and recognized limitations, it is clear that improving EV battery health requires a more nuanced approach that considers not just standard drive cycle metrics, but the complex interplay of average speed, resting periods, charging behaviors, and real-world conditions such as terrain and temperature. In conclusion, this work uncovers the complex dynamics that dictate EV battery aging, shifting the focus from singular metrics to a broader understanding of usage patterns and environmental factors. It underscores the importance of balanced driving habits, thoughtful charging strategies, and consideration of climate impacts as integral to enhancing battery longevity and overall vehicle performance. This comprehensive analysis informs OEMs on customizing driving patterns, charging scenarios, and rates to extend battery EOL, alleviate cost burdens, and ensure optimal performance over time. Stakeholders can optimize battery longevity based on user behavior, region-specific conditions, and driving patterns. This deeper understanding shatters common misconceptions and equips stakeholders with the knowledge needed to combat capacity fade under real-world driving conditions.

Future work should expand testing to include a wider variety of region-specific drive patterns, particularly for routes with significant road gradients and mountainous terrains, to better account for additional traction and power demands. Incorporating these conditions will help develop more realistic models for predicting battery aging and support strategies that optimize driving patterns to balance energy use and rest periods, ultimately mitigating unnecessary capacity fade. Additionally, adapting charging strategies to better reflect real-world user behavior is crucial. Integrating smart charging protocols that can manage charging interruptions, optimize charge rates, and adjust for ambient temperature could significantly reduce harmful effects like lithium plating and excessive SEI growth. Further research should focus on dynamic charging management, possibly aided by onboard predictive algorithms that adapt to user routines and local climate conditions. Combined with the adaptable framework demonstrated in this study, such advancements can pave the way for more practical, durable, and user-friendly battery systems that maintain health and performance throughout the EV's lifetime.

## Conflicts of interest

There are no conflicts to declare.

## Abbreviations

*a*_C_
: Characteristic acceleration
*v*_aerodynamic_
: Aerodynamic speed
*v*_*i*_
: Vehicle speed at *i*th instant
*g*
: Acceleration due to gravity
*h*
: Elevation
Δ*t*: Successive time interval
*T*
: Total time spent for driving
*S*
: Total distance travelled
m_Li_: Total moles of usable lithium
*θ* ^−^
: Volume-averaged particle concentrations of negative electrode
*θ* ^+^
: Volume-averaged particle concentrations of positive electrode
*C* ^−^
: Charge capacity of the negative electrode
*C* ^+^
: Charge capacity of the positive electrode
*C* ^e^
: Electrode charge capacity
*ε* ^e^
: Electrode active material volume fraction
*A*_c_
: Surface area of the current collector
*L* ^e^
: Electrode thickness
*c* ^e^
: Electrode molar concentration
EV: Electric vehicle
EOL: End-of-life
OEM: Original equipment manufacturer
WLTC: Worldwide harmonized light vehicles test cycle
UDDS: Urban dynamometer driving schedule
NEDC: New European driving cycle
IDC: Indian driving cycle
KI: Kinetic intensity
RPA: Relative positive acceleration
PKE: Positive kinetic energy
SEI: Solid electrolyte interphase
ICE: Internal combustion engine
SOC: State of charge
DS: Daily scenario
LLI: Loss of lithium inventory
LAM: Loss of active material
NMC: Nickel manganese cobalt
EC: Ethylene carbonate
EMC: Ethyl methyl carbonate
OCV: Open circuit voltage
GITT: Galvanostatic intermittent titration technique
CBD: Carbon and binder domains
EDS: Energy-dispersive X-ray spectroscopy
ICP-OES: Inductively coupled plasma optical emission spectroscopy
CV: Constant voltage
CC: Constant current
CCCV: Constant current constant voltage

## Data Availability

The datasets supporting this article have been uploaded as part of the SI. Further, the drive cycle data and the code for EOL-Assessment-Framework can be accessed *via* the link https://github.com/mlnsai/EOL-Assessment-Framework.
